# Supplementary material for: Effect of weight loss on the retinochoroidal structural alterations among patients with exogenous obesity
Source: PLoS One. 2020 Jul 9;15(7):e0235926. doi: 10.1371/journal.pone.0235926 (PMC7347179; doi:10.1371/journal.pone.0235926)
Supplement: Attachment — Submitted filename: PONE-D-20-08014_R1 tracked.pdf [file pone.0235926.s003.pdf]

PLOS ONE

Effect of Weight Loss on the Retinochoroidal Structural Alterations Among Patients with Exogenous Obesity

--Manuscript Draft--

**Manuscript Number:**	PONE-D-20-08014R1
**Article Type:**	Research Article
**Full Title:**	Effect of Weight Loss on the Retinochoroidal Structural Alterations Among Patients with Exogenous Obesity
**Short Title:**	Retinochoroidal vascularity in obesity
**Corresponding Author:**	Vishali Gupta, M.D.<br>Post Graduate Institute of Medical Education and Research<br>Chandigarh, INDIA
**Keywords:**	Optical coherence tomography; choroidal vascularity index; obesity; bariatric surgery; choroid; Perfusion
**Abstract:**	Purpose: To evaluate the changes in the retinochoroidal vasculature in patients with exogenous obesity using swept-source optical coherence tomography (SS-OCT) and OCT angiography (OCTA).<br>Methods: In this prospective study, 60 patients diagnosed with obesity (47 males) (mean age: 46.47±10.9 years) were included, of which 30 patients underwent bariatric surgery (Group A), and 30 patients underwent conservative management (exercise/diet) (Group B). Parameters including choroidal thickness (CT), choroidal vascularity index (CVI) and retinal capillary density index (CDI) and arteriovenous ratio (AVR) were measured at the baseline and three months follow up. 30 eyes (30 age and gender-matched) of normal subjects were included for comparison.<br>Results: Baseline CT was lower in 60 subjects with obesity compared to controls. Compared with normal subjects, subjects with obesity had higher mean CVI (0.66±0.02 versus 0.63±0.04; p<0.01), smaller FAZ area (0.26±0.07 versus 0.45±0.32; p<0.01), higher CDI (superficial plexus: 0.7±0.04 versus 0.68±0.06; p=0.04, deep plexus: 0.38±0.02 versus 0.35±0.06; p=0.01), and lower AVR (0.68±0.05 versus 0.70±0.03 versus; p<0.01). At 3-month after intervention, CT showed a significant increase in subjects from Group A (329.27±79μm; p<0.01) but not in Group B from baseline. No significant change was noted in CVI or CDI at 3-month in either group compared to baseline. AVR significantly increased in Group B (p=0.03).<br>Conclusion: Subclinical changes in retinochoroidal vasculature occurs in subjects with exogenous obesity compared to healthy subjects. Surgical intervention (bariatric surgery) may have a favorable outcome on the choroidal thickness in these patients.<br>Purpose: To evaluate the changes in the retinochoroidal vasculature in patients with exogenous obesity using swept-source optical coherence tomography (SS-OCT) and OCT angiography (OCTA).<br>Methods: In this prospective study, 60 patients diagnosed with obesity (47 males) (mean age: 46.47±10.9 years) were included, of which 30 patients underwent bariatric surgery (Group A), and 30 patients underwent conservative management (exercise/diet) (Group B). Parameters including choroidal thickness (CT), choroidal vascularity index (CVI) and retinal capillary density index (CDI) and arteriovenous ratio (AVR) were measured at the baseline and three months follow up. 30 eyes (30 age and gender-matched) of normal participants were included for comparison.<br>Results: Baseline CT was lower in 60 participants with obesity compared to controls. Compared with normal subjects, subjects with obesity had higher mean CVI (0.66±0.02 versus 0.63±0.04; p<0.01), smaller FAZ area (0.26±0.07 versus 0.45±0.32; p<0.01), higher CDI (superficial plexus: 0.7±0.04 versus 0.68±0.06; p=0.04, deep plexus: 0.38±0.02 versus 0.35±0.06; p=0.01), and lower AVR (0.68±0.05 versus 0.70±0.03 versus; p<0.01). At 3-month after intervention, CT showed a significant increase in participants from Group A (329.27±79μm; p<0.01) but not in Group B from baseline. No significant change was noted in CVI or CDI at 3-month in either group compared to baseline. AVR significantly increased in Group B (p=0.03).<br>Conclusion: Subclinical changes in retinochoroidal vasculature occurs in patients with exogenous obesity compared to healthy subjects. Surgical intervention (bariatric surgery) may have a favorable outcome on the choroidal thickness in these patients.

Powered by Editorial Manager® and ProduXion Manager® from Aries Systems Corporation

**Order of Authors:**	Aniruddha Agarwal
	Arshiya Saini
	Sarakshi Mahajan
	Rupesh Agrawal
	Carol Y. Cheung
	Ashu Rastogi
	Rajesh Gupta
	Yu Meng Wang
	Vishali Gupta, M.D.
	Michael Kwan
**Opposed Reviewers:**	
**Response to Reviewers:**	May 11, 2020<br><br>Pukhraj Rishi, MD<br>Academic Editor<br>PLOS ONE<br><br>RE: PONE-D-20-08014: Effect of Weight Loss on the Retinochoroidal Structural Alterations Among Patients with Exogenous Obesity<br><br>Dear Dr. Rishi:<br><br>On behalf of all the authors, I wish to thank you and the reviewers helping us improve our manuscript. We appreciate the comments and have addressed them as indicated below.<br><br>Journal Requirements:<br>1. Please ensure that your manuscript meets PLOS ONE's style requirements, including those for file naming. The PLOS ONE style templates can be found at https://journals.plos.org/plosone/s/file?id=wjVg/PLOSOne_formatting_sample_main_body.pdf and https://journals.plos.org/plosone/s/file?id=ba62/PLOSOne_formatting_sample_title_authors_affiliations.pdf<br>Response 1: Thank you very much for providing the links to the PLOS ONE style templates. We have incorporated these in the revised manuscript.<br><br>2. Thank you for stating the following financial disclosure:<br>"The funders had no role in study design, data collection and analysis, decision to publish, or preparation of the manuscript"<br>At this time, please address the following queries:<br>a) Please clarify the sources of funding (financial or material support) for your study. List the grants or organizations that supported your study, including funding received from your institution.<br>b) State what role the funders took in the study. If the funders had no role in your study, please state: “The funders had no role in study design, data collection and analysis, decision to publish, or preparation of the manuscript.”<br>c) If any authors received a salary from any of your funders, please state which authors and which funders.<br>d) If you did not receive any funding for this study, please state: “The authors received no specific funding for this work.”<br>Please include your amended statements within your cover letter; we will change the online submission form on your behalf.<br>Response 2: We have noted the above instructions. We have not received any funding for this work. The statement, “The authors received no specific funding for this work” has been added to the Cover Letter. Thank you.<br><br>3. One of the noted authors is a group or consortium 'OCTA Study Group'. In addition

to naming the author group, please list the individual authors and affiliations within this group in the acknowledgments section of your manuscript. Please also indicate clearly a lead author for this group along with a contact email address.
Response 3: Thank you very much for the comment. As per the journal requirements, we have stated the details of the OCTA study group in the acknowledgements section of the manuscript. Individual email addresses are also listed.

4. Your ethics statement must appear in the Methods section of your manuscript. If your ethics statement is written in any section besides the Methods, please move it to the Methods section and delete it from any other section. Please also ensure that your ethics statement is included in your manuscript, as the ethics section of your online submission will not be published alongside your manuscript.
Response 4: Thank you very much for the comment. We have added the following statement in the methods section, "The study was approved by the Institute Ethics Committee of PGIMER and adhered to the tenets of Declaration of Helsinki."
Page 8, First Paragraph

Additional Editor Comments:
The reviewers have raised several pertinent concerns including those related to the methodology of the study. However, in view of the interesting research question, it is worthwhile to have the authors an opportunity to revise the manuscript.
Response: We would like to thank the Additional Editor for thoroughly reviewing our manuscript, and giving us the chance to respond to the reviewer's comments. We have significantly modified the manuscript based on the comments, clarified several aspects of the methodology, and improved reporting of the results. We hope that the revisions are appropriate, and the manuscript can be considered further.

Reviewer #1:
1. Table 1 details baseline characteristics of Groups A and B but not the normal subjects.
Response 1: Thank you very much for the comment. We have included baseline characters of normal control population in Table 1.

2. Table 2 does not give the standard deviations or confidence intervals.
Response 2: Thank you very much for the suggestions. We have included the standard deviation values in the Table 2.

3. The authors defined the obesity as BMI>27.5 and waist circumference of more than 80 cm. However, the authors do not give the actual distribution and comparison of the BMI and waist circumference between normal and obese subjects in this study. This may be important to make sure that the normal subjects are really normal.
Response 3: Thank you very much for highlighting this important aspect. We have mentioned the height, weight, BMI and waist circumference of the normal control subjects included in this study in the results section. The normal subjects had a mean BMI of 24.90 and mean waist circumference of 76.07 cm.

Results section; Page 13, 1st paragraph

4. The methodology does not specify where the choroidal thickness and retinal thickness were measured - in relation to fovea and whether the measurement taken for analysis is an average of several measurements?
Response 4: Thank you once again for highlighting this pertinent aspect. We have added the missing information in the methods. The measurements were performed at the fovea. The following statement has been added, "The measurements were performed at the fovea by two independent graders. The average of the two graders' measurements was used for analysis."

Methods section; Page 10; 2nd paragraph

5. One is not clear also whether the measurements within one subject were taken at roughly the same time of the day? There are studies to show significant diurnal variation in choroidal thickness of up to 33.7 micrometers (Tan CS, Ouyang Y, Ruiz H, Sadda SR. Diurnal variation of choroidal thickness in normal, healthy subjects measured by Spectral domain optical coherence tomography. IOVS 2012;53:261-266)

Powered by Editorial Manager® and ProduXion Manager® from Aries Systems Corporation

Response 5: As the reviewer suggested, it is important to keep in mind the diurnal variation of the choroidal thickness and therefore, all measurements need to be performed at a particular time interval in the day. In our study, all the measurements were performed between 10.00 am to 12.00 noon. This is also the common working hours of the Obesity Clinic and Ophthalmology Clinic in our institute. We have mentioned this information in the revised manuscript.

Page 9; Section on "Acquisition of Images"

6. Are there any obese subjects without comorbidities? They would form a good subgroup for analysis of effect of obesity itself (if any) on the parameters studied.
Response 6: In our cohort, among the patients in Group A (those undergoing surgery), there were only 2 patients who did not have additional co-morbidities. Likewise, in Group B (those receiving conservative therapy), 3 patients did not have any additional co-morbidities. Therefore, we believe that this is a small number that may not allow meaningful statistical analysis. Unfortunately, subjects with obesity tend to have various comorbidities, and this has been mentioned in the limitations of the manuscript.

Page 19, Last line; Page 20, first 3 lines

7. To evaluate the role of coexisting vascular disorder versus only obesity on the vascular parameters that have been studied, one would ideally need two controls- a) non-obese subjects with systemic vascular disorder (roughly of the same duration and severity as seen in the obese subjects. And b) as mentioned above- any subgroup of obese subjects without vascular comorbidity (perhaps such a group may be difficult to get).
Response 7: Thank you very much for the insightful comment. We agree with the reviewer that it would be ideal to have two such groups – one of non-obese subjects with systemic vascular diseases of same duration and severity, and obese subjects without vascular comorbidity. But as the reviewer suggests, in the real world, it is very difficult to obtain such a cohort especially because some of these diseases co-exist. We have added this factor in the discussion, highlighting the limitations of our selection.

Page 20; First paragraph

8. The authors conclude – 'While the choroidal thickness increased significantly in subjects undergoing weight reduction surgery, there was no change in the vascular parameters such as CVI, CDI and FAZ area, indicating that these vascular alterations may be irreversible.' There are several issues to this sweeping conclusion.
a) At baseline the authors found choroidal thickness and retinal thickness to be similar between normal subjects and obese subjects. But the obese subjects had increased choroidal thickness after bariatric surgery - does this mean their choroidal thickness is now supra normal?
b) The other vascular parameters - CVI, AVR, FAZ, CDI have not altered before and after. To conclude this- as indicative of permanent damage to vessels would also force one to admit that the choroidal thickness becomes supra normal after bariatric surgery!
c) The authors excluded all patients with obvious retinal vascular disorder among the subjects with obesity. Hence it is understandable that the systemic vascular disease is also likely to be of short duration and perhaps under adequate control.
Under such circumstances it would be difficult to accept a conclusion that attributes the lack of difference in the parameters before and after weight reduction to irreversible vascular changes.
Response 8: Thank you very much for the comments. We agree with your observations and accordingly, have performed additional literature search to understand the choroidal flow and autoregulation in systemic diseases. In response to point b, we also calculated Mean ocular perfusion pressure (MOPP) as one of the vascular flow regulatory factor in the ocular circulation and possibly affecting CVI, AVR and CDI. The revised manuscript now provides an explanation as to why the choroidal thickness could have increased following surgery.

We observed an interesting finding in our study. The mean arterial pressure (MAP) significantly reduced in Group A (those subjects undergoing surgery) and not in Group B (patients managed conservatively). However, there was no statistically significant

Powered by Editorial Manager® and ProduXion Manager® from Aries Systems Corporation

difference in MOPP in both the groups. This has been added to the revised Table 2. Studies have shown that the systemic MAP reduces both in the long-term and short-term in response to bariatric surgery. Choroidal blood flow is significantly affected by MAP, and therefore, it may result in increased choroidal thickness postoperatively in patients of Group A. Hence, the choroidal thickness should not be labeled as 'supra normal'.

Among the other vascular parameters, CVI and CDI were lower in obese subjects compared to normal healthy controls. However, we have removed the argument that change in CVI and CDI indicates permanent damage to these structures.

Page 19, multiple places

Reviewer #2:
1. The comparison of baseline features in subjects versus controls is skewed by significant prevalence of diabetes and hypertension, etc. which are known to affect choroidal vasculature and FAZ. The groups A, B are different systemically - when systemic disease and treatment is being studied for its effect on an ocular finding, we expect the groups to be better matched.
Response 1: We would like to thank the reviewer for the insightful comments. The baseline comorbidities are different among subjects in Groups A and B. This is likely due to a selection bias, i.e. subjects who are in Group A and undergoing bariatric surgery may be the ones with higher BMI (as reflected in Table 2), and with higher BMI, complications and comorbidities such as hypertension and sleep apnea can be higher than those in Group B (patients not undergoing surgery). Therefore, this has been mentioned in the revised manuscript in the limitations.
However, the variability of the comorbidities and systemic disease between Group A and Group B will not affect the overall comparison of patients (both groups combined) with normal subjects, nor will it affect the before-after comparison of subjects within the groups. Therefore, we believe that our data set is still pertinent and with the inherent limitations, comparison between the groups is still possible. Thank you very much.

Page 20; Discussion

2. Also, we do not know what happened to the systemic disease after bariatric surgery or weight reduction - did it affect blood pressures, DM control, etc?
Response 2: Thank you very much for the comment. We have already reported the follow-up changes in the weight, BMI, waist circumference, and HB1AC in patients from Group A and B in Table 2. Additionally, we have added the mean values of systolic and diastolic blood pressures in the two groups at baseline and follow-up in Table 2. We also calculated and added the systemic mean arterial pressure (MAP) in Table 2, and realized that subjects undergoing bariatric surgery (Group A) had a significantly reduced MAP in the follow-up period.

In the follow-up, the subjects also experienced a statistically significant improvement in parameters such as weight (in Group A), BMI, waist circumference and HB1AC. However, there was no statistically significant difference in the mean systolic and diastolic blood pressure. This information is provided in Table 2.

3. We know that HTN/ DM get better with weight reduction and more so with bariatric surgery... so the systemic status of the groups and changes in medication after the intervention will be useful to know, and any meaningful conclusion can only be derived from that.
Response 3: Thank you very much for the comment. In Group A (the subjects who underwent bariatric surgery), the systolic and diastolic blood pressure improved at follow-up, but did not reach statistical significance. However, there was improvement in diabetes (reflected by HB1AC values). Thank you very much for the suggestion.

Results; Table 2

4. Where there are so many systemic variables confounding the basic disease (Obesity), the study would need a much larger sample size to have relevant statistics.
Response 4: The sample size has been calculated for the study based on the study by Dogan et al [26] considering equal allocation ratio of 1:1, which will provide the desired

effect with 95% confidence and 80% power. As per Dogan et al, the choroidal thickness for obese group was 300.63 ± 65.55 μm, while that of normal group was 338.79 ± 64.41 μm. Accordingly, the effect size obtained was 0.59 (~0.60). In the current study, a sample size of in each group was obtained as 30. However, we do agree that a larger data set would help us understand the changes in the parameters better. Large dataset is challenging to obtain, since the number of patients undergoing bariatric surgery may be less. Hence, in the discussion, we have stated that one of the limitations of our study is the modest sample size.

Methods Section; Page 12; 2nd paragraph

Reviewer #3:
1. Obesity is a female preponderant disease. How do you explain male preponderance in your cases? Was diurnal variation accounted for while measuring choroidal thickness? Co-morbidities are mentioned at baseline but not at final follow up. What improvements were noted in co-morbid factors like BP, Hb AIC at final follow up and their effect on final CT and other variables?
Response 1: Thank you very much for highlighting these pertinent aspects. We agree that obesity is a female preponderant disease. However, in India, gender disparities in health care expenditures are well known due to various socioeconomic factors. Studies have shown that the health care expenditures on adult Indian females is systemically lower than that of adult males. Such socioeconomic disparities in health and health care are major concerns in India. We have added these references and the possible selection bias in the limitations.
We performed the OCT scans between 10.00 am to 12.00 noon in our study to minimize the effect of diurnal variations (explained in the response to reviewer #1, comment 5).
We have now mentioned the comorbidities at follow-up as well. The improvements in HB1AC, BP and mean arterial pressure has been added to the revised manuscript.

Page 20; Discussion; Table 2; multiple places

2. CT depends on multitude of factors. Was multivariate analysis performed to look for the effect of other variables? Kindly provide the data

Response 2: Thank you very much for raising this very valid point. We have also added Mean ocular perfusion pressure (MOPP) in addition to all the possible factors which can affect CT and other vascular parameters in the eye. We have included the multiple regression analysis in the revised results. The multiple regression reveals that none of the confounders had a significant impact on the change in choroidal thickness from baseline to 3 months. It is noteworthy that with every unit increase in HB1AC, there was a change in the choroidal thickness of approximately 12 microns, but this was not statistically significant. A limitation, therefore, has been added to the discussion that a higher sample would be better for a detailed analysis.
An interesting aspect was that the only variable associated with higher choroidal thickness on multivariate analysis was the treatment strategy (significantly higher in those undergoing bariatric surgery).

Page 15; paragraph 3

3. Review of literature reveals that CT is higher in patients with obesity. However the present study shows decreased CT in patients with obesity. This appears to be major conflicting result vis a vis published literature. Authors need to clarify this point in a more robust way in review of literature
1. Yilmaz I, Ozkaya A, Kocamaz M, et al. CORRELATION OF CHOROIDAL THICKNESS AND BODY MASS INDEX. Retina (Philadelphia, Pa). 2015;35(10):2085-2090. doi:10.1097/IAE.0000000000000582

2. Ophthalmic Surg Lasers Imaging Retina. 2017 Jan 1;48(1):10-17. doi: 10.3928/23258160-20161219-02. Choroidal Thickness in Childhood Obesity. Bulus AD, Can ME, Baytaroglu A, Can GD, Cakmak HB, Andiran N.

3. BMC Ophthalmol. 2016 May 4;16(1):48. doi: 10.1186/s12886-016-0227-z. Choroidal thickness in obese women. Yumusak E1, Ornek K2, Durmaz SA3, Cifci A4,

Powered by Editorial Manager® and ProduXion Manager® from Aries Systems Corporation

	Guler HA2, Bacanli Z4.<br>Response 3: Thank you very much for the comment. We agree that the previous studies listed by the reviewer have reported a higher choroidal thickness in subjects with obesity compared to healthy controls. In our study, the choroidal thickness in normal subjects was 278.9 microns, and 284.2 microns in subjects with obesity. Thus, we also found higher choroidal thickness values compared to normal subjects, but this did not reach statistical significance.<br><br>The manuscript by Yilmaz et al showed that subjects with BMI between 18.5-24.9 have higher choroidal thickness than those with BMI > 25. In the study by Bulus et al, the choroidal thickness was higher in obese children. Yumusak et al have also shown higher choroidal thickness values in obese women compared to control population. The revised statements and newer references have been added in the revised manuscript.<br><br>Page 17; Paragraph 2<br><br>Author Changes<br>We have added the values for mean ocular perfusion pressure (MOPP) calculated from mean arterial pressure and IOP. This has been mentioned in the methods (Page 9; first paragraph) and results (Page 13; 2nd paragraph).<br><br>We hope our responses will satisfy the reviewers. We thank the Editor and the reviewers for dedicating their time and effort to review our manuscript. We hope that the manuscript is now appropriate for publication in Retina.<br><br>Sincerely,<br><br>Vishali Gupta, MS for the authors
**Additional Information:**	
**Question**	**Response**
**Financial Disclosure**<br><br>Enter a financial disclosure statement that describes the sources of funding for the work included in this submission. Review the submission guidelines for detailed requirements. View published research articles from *PLOS ONE* for specific examples.<br><br>This statement is required for submission and **will appear in the published article** if the submission is accepted. Please make sure it is accurate.	The authors received no specific funding for this work.

**Unfunded studies**<br>Enter: *The author(s) received no specific funding for this work.*<br><br>**Funded studies**<br>Enter a statement with the following details:<br>• Initials of the authors who received each award<br>• Grant numbers awarded to each author<br>• The full name of each funder<br>• URL of each funder website<br>• Did the sponsors or funders play any role in the study design, data collection and analysis, decision to publish, or preparation of the manuscript?<br>• **NO** - Include this sentence at the end of your statement: *The funders had no role in study design, data collection and analysis, decision to publish, or preparation of the manuscript.*<br>• **YES** - Specify the role(s) played.<br><br>* typeset	
**Competing Interests**<br><br>Use the instructions below to enter a competing interest statement for this submission. On behalf of all authors, disclose any competing interests that could be perceived to bias this work—acknowledging all financial support and any other relevant financial or non-financial competing interests.<br><br>This statement **will appear in the published article** if the submission is accepted. Please make sure it is accurate. View published research articles from *PLOS ONE* for specific examples.	The authors have declared that no competing interests exist

Powered by Editorial Manager® and ProduXion Manager® from Aries Systems Corporation

**NO authors have competing interests**<br><br>Enter: *The authors have declared that no competing interests exist.*<br><br>**Authors with competing interests**<br><br>Enter competing interest details beginning with this statement:<br><br>*I have read the journal's policy and the authors of this manuscript have the following competing interests: [insert competing interests here]*<br><br>* typeset	
**Ethics Statement**<br><br>Enter an ethics statement for this submission. This statement is required if the study involved:<br><br>• Human participants<br>• Human specimens or tissue<br>• Vertebrate animals or cephalopods<br>• Vertebrate embryos or tissues<br>• Field research<br><br>Write "N/A" if the submission does not require an ethics statement.<br><br>General guidance is provided below. Consult the submission guidelines for detailed instructions. **Make sure that all information entered here is included in the Methods section of the manuscript.**	Since the study involved human subjects, we obtained clearance from Ethics Committee of Post Graduate Institute of Medical Education and Research (PGIMER), Chandigarh, India. The approval number is PGI/NK/2019/035.

**Format for specific study types**<br><br>**Human Subject Research (involving human participants and/or tissue)**<br>• Give the name of the institutional review board or ethics committee that approved the study<br>• Include the approval number and/or a statement indicating approval of this research<br>• Indicate the form of consent obtained (written/oral) or the reason that consent was not obtained (e.g. the data were analyzed anonymously)<br><br>**Animal Research (involving vertebrate animals, embryos or tissues)**<br>• Provide the name of the Institutional Animal Care and Use Committee (IACUC) or other relevant ethics board that reviewed the study protocol, and indicate whether they approved this research or granted a formal waiver of ethical approval<br>• Include an approval number if one was obtained<br>• If the study involved *non-human primates*, add *additional details* about animal welfare and steps taken to ameliorate suffering<br>• If anesthesia, euthanasia, or any kind of animal sacrifice is part of the study, include briefly which substances and/or methods were applied<br><br>**Field Research**<br><br>Include the following details if this study involves the collection of plant, animal, or other materials from a natural setting:<br>• Field permit number<br>• Name of the institution or relevant body that granted permission	
**Data Availability**<br><br>Authors are required to make all data underlying the findings described fully available, without restriction, and from the time of publication. PLOS allows rare exceptions to address legal and ethical concerns. See the PLOS Data Policy and FAQ for detailed information.	Yes - all data are fully available without restriction

A Data Availability Statement describing where the data can be found is required at submission. Your answers to this question constitute the Data Availability Statement and **will be published in the article**, if accepted.<br><br>**Important:** Stating 'data available on request from the author' is not sufficient. If your data are only available upon request, select 'No' for the first question and explain your exceptional situation in the text box.<br><br>Do the authors confirm that all data underlying the findings described in their manuscript are fully available without restriction?	
**Describe where the data may be found in full sentences. If you are copying our sample text, replace any instances of XXX with the appropriate details.**<br><br>• If the data are **held or will be held in a public repository**, include URLs, accession numbers or DOIs. If this information will only be available after acceptance, indicate this by ticking the box below. For example: *All XXX files are available from the XXX database (accession number(s) XXX, XXX.).*<br>• If the data are all contained **within the manuscript and/or Supporting Information files**, enter the following: *All relevant data are within the manuscript and its Supporting Information files.*<br>• If neither of these applies but you are able to provide **details of access elsewhere**, with or without limitations, please do so. For example:<br><br>*Data cannot be shared publicly because of [XXX]. Data are available from the XXX Institutional Data Access / Ethics Committee (contact via XXX) for researchers who meet the criteria for access to confidential data.*<br><br>*The data underlying the results presented in the study are available from (include the name of the third party*	All relevant data are within the manuscript and its Supporting Information files.

and contact information or URL).<br>• This text is appropriate if the data are owned by a third party and authors do not have permission to share the data.<br><br>* typeset	
Additional data availability information:	

Dr. Vishali Gupta

Professor in Ophthalmology

Post Graduate Institute of Medical Education and Research, Chandigarh, India

pgimer.edu.in

To
The Editor-in-chief,
PLOS One

Subject: Submission of manuscript for publication

Dear Editor,

On behalf of all the authors we are submitting a manuscript '*Effect of Weight Loss on the Retinochoroidal Structural Alterations Among Patients with Exogenous Obesity*' for kind consideration for publication.

Based on the suggestions of the editorial board and the reviewers, we have made substantial changes to the manuscript. Thus, we hope to have addressed the major concerns and that the manuscript is now suitable for publication. We greatly appreciate your help in this regard.

We have attached the revised version along with the responses to reviewers' comments document. Please let us know if you have any questions.

Thank you for your consideration of our manuscript. Please let us know if we can provide you with any additional information. We look forward to hearing from you.

Funding: The authors received no specific funding for this work.

Sincere Regards,

Vishali Gupta, MD
vishalisara@yahoo.co.in

Manuscript

Click here to access/download;Manuscript;Obesity_CVI_01242020_AA_Final_

Title: Effect of weight loss on the retinochoroidal structural alterations among patients with exogenous obesity

Authors: Aniruddha Agarwal, MS,[1] Arshiya Saini, MBBS,[1] Sarakshi Mahajan, MD,[2] Rupesh Agrawal, FRCS,[3] Carol Y. Cheung, PhD,[4] Ashu Rastogi, MD,[5] Rajesh Gupta, MS,[6] Yu Meng Wang, PhD,[4] Michael Kwan, MD[4] Vishali Gupta, MS[1*] for the OCTA Study Group^

Affiliations:

1: Advanced Eye Center, Department of Ophthalmology, Post Graduate Institute of Medical Education and Research (PGIMER), Chandigarh, India

2: School of Medicine, St Joseph Mercy Hospital, Oakland, Pontiac, Michigan (USA)

3: National Healthcare Group Eye Institute, Department of Ophthalmology, Tan Tock Seng Hospital, Singapore

4: Department of Ophthalmology and Visual Sciences, The Chinese University of Hong Kong, Hong Kong

5: Department of Endocrinology, Post Graduate Institute of Medical Education and Research (PGIMER), Chandigarh, India

6: Department of General Surgery, Post Graduate Institute of Medical Education and Research (PGIMER), Chandigarh, India

^ Membership of the OCTA study group is provided in the acknowledgements

Short Title: Retinochoroidal vascularity in obesity

Corresponding Author

* Vishali Gupta, MS (vishalisara@yahoo.co.in, vishalisara@gmail.com)

SUMMARY STATEMENT

Exogenous obesity is linked to various systemic and ocular pathologies. Using swept-source optical coherence tomography and optical coherence tomography angiography, we observed altered retinochoroidal vasculature in participants with exogenous obesity compared to healthy control participants. These parameters showed improvement following interventions such as bariatric surgery.

Abstract

Purpose: To evaluate the changes in the retinochoroidal vasculature in patients with exogenous obesity using swept-source optical coherence tomography (SS-OCT) and OCT angiography (OCTA).

Methods: In this prospective study, 60 patients diagnosed with obesity (47 males) (mean age: 46.47±10.9 years) were included, of which 30 patients underwent bariatric surgery (Group A), and 30 patients underwent conservative management (exercise/diet) (Group B). Parameters including choroidal thickness (CT), choroidal vascularity index (CVI) and retinal capillary density index (CDI) and arteriovenous ratio (AVR) were measured at the baseline and three months follow up. 30 eyes (30 age and gender-matched) of normal participants were included for comparison.

Results: Baseline CT was lower in 60 participants with obesity compared to controls. Compared with normal subjects, subjects with obesity had higher mean CVI (0.66±0.02 versus 0.63±0.04; $p<0.01$), smaller FAZ area (0.26±0.07 versus 0.45±0.32; $p<0.01$), higher CDI (superficial plexus: 0.7±0.04 versus 0.68±0.06; $p=0.04$, deep plexus: 0.38±0.02 versus 0.35±0.06; $p=0.01$), and lower AVR (0.68±0.05 versus 0.70±0.03 versus; $p<0.01$). At 3-month after intervention, CT showed a significant increase in participants from Group A (329.27±79µm; $p<0.01$) but not in Group B from baseline. No significant change was noted in

CVI or CDI at 3-month in either group compared to baseline. AVR significantly increased in Group B (p=0.03).

Conclusion: Subclinical changes in retinochoroidal vasculature occurs in participants with exogenous obesity compared to healthy subjects. Surgical intervention (bariatric surgery) may have a favorable outcome on the choroidal thickness in these patients.

Keywords: Optical coherence tomography; choroidal vascularity index; obesity; bariatric surgery; choroid; perfusion

Introduction

Obesity is commonly caused by a combination of excessive food intake, lack of physical activity, and genetic susceptibility though some cases maybe be caused primarily by genetic predisposition, endocrine disorders, medications, or mental disorders, and is measured in terms of body mass index (BMI) [1,2]. Obesity may increase levels of some vasoconstrictor molecules such as endothelin-1 and angiotensin-II [3,4] and has been linked with various ocular diseases including glaucoma, diabetic retinopathy, cataract, and age-related macular degeneration [5–7]. Therefore, obesity may be associated with alterations in the retinal microvasculature and the choroid by a number of mechanisms, which may be the basis for the various ocular diseases.

Assessment of retinal microvasculature includes measuring diameter of retinal arterioles and venules from fundus photographs [8]. The arteriole-to-venule ratio (AVR) reflects preclinical changes in cerebral and coronary microcirculation. The AVR is calculated using the mean vessel calibre of the largest six arteries (central retinal arteriolar equivalent - CRAE) and largest six venules (central retinal venular equivalent - CRVE). In children with obesity, CRAE has been strongly linked to severe cardiovascular risk factors [9]. Similarly, CRAE has been negatively correlated to fat mass indices in children with obesity, reflecting presence of narrow arterioles in these patients affecting the retinal microcirculation [10]. Obesity also results in wider CRVE and lower AVR that correlate with higher leptin levels, indicating systemic metabolic abnormalities [11]. Improved AVR has been found in patients undergone bariatric surgery after 6 months [12] and 9 months of surgery [13].

Choroidal thickness has been found to be affected by physiological variations such as age, gender, refractive status, as well as several local and systemic diseases [14–16]. Literature shows that the choroidal thickness is lower in patients with BMI>25.0 compared to patients with BMI between 18-24 [17]. Recently, availability of swept-source optical coherence tomography (SS-OCT) and optical coherence tomography angiography (SS-OCTA) makes it possible to have precise measurements including choroidal thickness and vascular density indices [18]. The newer indicator of measuring choroidal vascularity index (CVI) indicates the ratio of choroidal vessels to stroma thus allowing the more accurate insight into the vascular alterations associated with various systemic or local diseases [19]. CVI has been reported to decrease in patients with age-related macular degeneration [20] and intraocular tuberculosis [21], while an increase is noted in central serous chorioretinopathy [22].

The alterations caused by obesity, if any, can be quantified by measuring retinal and choroidal thickness, CVI, and AVR. There is a paucity of studies in the literature that have determined the such retinochoroidal alterations in details. Moreover, the effect of correction of obesity by bariatric surgery or non-surgical measures on retinochoroidal vasculature has not been evaluated. The present study aims to describe the quantitative retinochoroidal vascular alterations associated with obesity at baseline and its correlation following an intervention (bariatric surgery or conservative management).

Materials and Methods

The index study was a prospective observational study that included 60 adult patients of either gender with exogenous obesity who presented to Department of Endocrinology, Postgraduate Institute of Medical Education and Research (PGIMER), Chandigarh. Patients who fulfilled the following inclusion criteria were enrolled in the study: Age 18-70 years, BMI≥27.5 with exogenous obesity, waist circumference of ≥80 cm, patients willing to undergo weight reduction (either medical or surgical measures). The exclusion criteria were patients with endogenous obesity (such as Cushing's syndrome), patients with retinal pathologies that can affect the retinal/choroidal thickness or vascularity such as diabetic retinopathy, macular degeneration, central serous chorioretinopathy, optic atrophy, glaucoma, and uveitis. Patients where media clarity is obscured by the presence of cataract, vitritis or any other such co-existent pathology that does not allow the acquisition of good images were also excluded. In addition, patients with refractive errors more than +3 or -3 D (high hypermetropia and myopia) were also excluded. The study was approved by the Institute Ethics Committee of PGIMER. Written informed consent was obtained from the patients enrolled in the study. The study adhered to the tenets of the Declaration of Helsinki and the provisions of the Health Insurance Portability and Accountability Act.

Baseline Evaluation

Detailed history regarding associated co-morbidities including diabetes mellitus, hypertension, obstructive sleep apnea, osteoarthritis was recorded. A detailed treatment history regarding concomitant medications being taken by the patient for several associated co-morbidities was recorded. A general physical examination was done and parameters including

height, weight, BMI, waist index, blood pressure were done. The mean arterial pressure (MAP) was also calculated. The best-corrected visual acuity (BCVA) by Snellen's chart (converted to LogMAR units for statistical analysis), intraocular pressure (IOP) measured by non-contact tonometer, slit lamp biomicroscopy for anterior segment and posterior segment examination were performed. Using the MAP and IOP values, the mean ocular perfusion pressure (MOPP) was also calculated as 2/3 of the difference between MAP and IOP.

Acquisition of Images

Thirty-degree color fundus photographs were acquired on the digital fundus camera (DRI Triton, Topcon®) with images focussed on disc and macula. SS-OCT 3D and 5-Line raster scan of the macula and optic disc were done. SS-OCTA (DRI Triton, Topcon®) using 3 × 3 scans of the macula and optic nerve head were performed. The acquisition of the scans were at least twice and better quality images were selected for further analysis. The acquisition of images was performed between 10.00 am to 12.00 noon in our study (common working hours of the Departments of Endocrinology Obesity Clinic and Ophthalmology). This also helps to minimize the effect of diurnal variations in choroidal thickness [16]. The acquired images were analyzed by three independent observers (AA, RA, and VG) and AVR, retinal and choroidal thickness, CVI, retinal thickness and retinal vascularity index were measured.

Measurement of Arteriovenous ratio

The AVR was calculated at the optic nerve head and macula using the fundus photographs. Retinal vascular caliber was measured following a standardized protocol, based on the revised Knudtson–Parr–Hubbard formula, as described in previous publications [23–25]. The CRAE and CRVE values were used to calculate the AVR for all the participants in the study.

Measurement of Retinal and Choroidal Thickness

Retinal thickness was measured from the inner border of the internal limiting membrane to the outer border of the retinal pigment epithelium (RPE). Choroidal thickness was measured vertically from the outer border of the RPE to the inner border of the sclera. The upper border was marked at the RPE and the lower border area was below the line of light pixels at the choroid-scleral junction. The measurements were performed at the fovea by two independent graders. The average of the two graders' measurements was used for analysis.

Measurement of CVI

In order to measure the CVI, image binarization was performed for all the scans obtained from patients. Subfoveal scan (central B-scan) was chosen for image analyses. The image was processed on public domain software Image J (National Institutes of Health, Bethesda, USA). Polygon tool was used to select the total choroid area (TCA), which was added in the region of interest (ROI) manager. After converting the image into 8 bit, Niblack auto local thresholding was subsequently applied which gave the mean pixel value with standard deviation for all the points. On the SS-OCT scans, the luminal Area (LA) was highlighted by applying the color

threshold. In order to determine the LA within the selected polygon, both the areas in ROI manager were selected and merged by ‘AND’ operation of Image J. The composite third area was added to the to the ROI manager. The first area represents the total of the choroid selected, and the third composite area is the vascular or LA. Stromal area (SA), which corresponds to the interstitial or stromal component of the choroid, was obtained by calculating the differences between TCA and LA. The CVI was calculated by dividing LA by TCA.

Measurement of Retinal Capillary network

The retinal capillary network was measured in terms of foveal avascular zone (FAZ) area and capillary density index (CDI). For calculation of FAZ, manual delineation was performed on a third-party software (ImageJ). The area was calculated in square millimetres. A circle with a radius of 1.5mm is centered at the subfoveal region. Using the Niblack thresholding and ROI manager, all images were binarized and converted to 8-bits with a mean pixel value and standard deviation of all points. Subsequently, the LA was highlighted within the circle with the brightness set to 0 and 254. The LA was merged with the corresponding threshold area and measured using ROI manager. The CDI was defined as the percentage of capillary density over the stromal area within the 1.5 mm-radius circle at the macula region. CDI was obtained at both, superficial and deep retinal capillary plexus.

The patients were followed-up at 3 months following intervention (conservative management that included diet therapy and exercise, or bariatric surgery). Patients who underwent surgery (n=30) were categorized into Group A, and patients receiving conservative

therapy (n=30) were categorized to Group B. At follow-up, they underwent ocular examination, fundus photography, SS-OCT and OCTA. All the above parameters were repeated at 3 months.

We included a normative database of 30 participants from our center with no known ocular or systemic disease (mean age: 33.6 ± 8.53 years; 21 males). Similar parameters, i.e. retinal and choroidal thickness, CVI, FAZ area, CDI and AVR were calculated, and compared to patients with obesity.

Statistical Analysis

We obtained the sample size by referring to the study by Dogan et al [26] considering equal allocation ratio of 1:1, which will provide the desired effect with 95% confidence and 80% power. The statistical comparison of parameters was carried out using non-parametric tests. Multivariate regression modelling were carried out to study the effect of confounders on choroidal thickness. All the analyses were performed using SPSS version 20.0 (IBM Inc.) and the statistical significance evaluated at 5% level.

Results

Study Participants, Demographics and Systemic Features

The study included a total of 60 patients with 30 patients in each group followed prospectively. The mean age of all patients was 46.47 ± 10.9 years (Table 1). There were a total

of 47 males in the study. Both the groups matched in their demographic profile at the baseline with no statistically significant difference in any parameter. The co-morbidities of the study participants and the specific treatments are mentioned in Table 1. The mean height, weight, waist circumference, blood pressure (including MAP), BMI, and other laboratory parameters including glycosylated hemoglobin (Hb1Ac) have been summarized in Table 2 (both at baseline and follow-up). At the end of 3 months, BMI was comparable in both the groups (36.50 ± 3.93 versus 39.68 ± 31.60; $p=0.59$). In the normal control participants, the mean height was 164.13 ± 12.3 cm, mean weight was 67.2 ± 10.64 kg (mean BMI: 24.90 ± 2.57) ($p<0.001$ compared to obese patients). The mean waist circumference in this group was 76.07 ± 3.76 cm ($p<0.001$ compared to obese patients).

In Group A, the mean BCVA was 0.007 ± 0.04 LogMAR units whereas it was 0.001 ± 0.01 LogMAR in Group B. There was no statistically significant change in the BCVA at the end of 3 months. In Group A, the MOPP was 55.78 mm Hg at baseline, and 55.01 mm Hg at 3 months ($p=0.07$), whereas in Group B, MOPP was 54.4 mm Hg at baseline, and 54.8 mm Hg at 3 months ($p=0.47$).

Comparison of Normal Participants with Obese Patients

In comparison to the normal participants (n=30), patients with obesity (n=60) did not have statistically significant differences in either the mean retinal or choroidal thickness values at baseline. However, the mean CVI was higher among normal subjects compared to participants with obesity (0.66 ± 0.02 versus 0.63 ± 0.04, respectively; $p<0.01$). The FAZ area was significantly lower in normal participants compared to patients with obesity (0.26 ± 0.07 versus

0.45 ± 0.32, respectively; $p<0.01$). CDI was also significantly higher in normal subjects compared to obese patients (superficial plexus: 0.70 ± 0.04 versus 0.67 ± 0.06, respectively; $p=0.04$, deep plexus: 0.38 ± 0.02 versus 0.35 ± 0.06, respectively; $p=0.01$). AVR was statistically higher in the normal subjects compared to patients with obesity (0.70 ± 0.03 versus 0.68 ± 0.05, respectively; $p<0.01$).

Table 3 compares the retinochoroidal parameters between normal participants and participants with obesity.

Baseline Image Analyses among Patients with Obesity in both Groups

The baseline retinal and choroidal thicknesses in patients from Group A are listed in Table 4. The mean CVI in Group A was 0.63 ± 0.04. Analyses of SS-OCTA among participants from Group A revealed a mean FAZ area of 0.42 ± 0.13 mm^2. The mean CDI among participants in Group A was 0.68 ± 0.06 in the superficial plexus and 0.36 ± 0.03 in the deep plexus. Finally, the AVR among the patients in Group A was 0.68 ± 0.06. The baseline and follow-up values of participants in Group B are listed in Table 5. The CVI measured 0.62 ± 0.04. The mean FAZ area was 0.48 ± 0.43 mm^2. The superficial and deep capillary plexus CDI was 0.66 ± 0.06 and 0.34 ± 0.08, respectively. AVR measured 0.67 ± 0.05.

Follow-up Image Analyses among Participants with Obesity

Follow-up values of patients of Group A and B are listed in Table 4 and 5. In Group A, the choroidal thickness showed a statistically significant increase at 3 months (329.27 ± 79; $p<0.01$). There was no significant change in the CVI among participants in Group A (0.61 ± 0.11; p=0.19). The follow-up measurements on SS-OCTA did not reveal any significant change in the FAZ area in subjects from Group A (0.43 ± 0.16 mm^2; p=0.99). Similarly, no change was noted in the CDI at 3 months in the superficial capillary plexus (0.69 ± 0.06; p=0.6) or the deep capillary plexus (0.37 ± 0.05; p=0.51). The AVR was higher at follow-up among subjects from Group A compared to baseline but not statistically significant (0.70 ± 0.05; p=0.06).

Follow-up measurements in Group B at 3 months revealed no significant change in either the retinal or choroidal thickness compared to baseline (Table 5). Similar to the observations in Group A, there were no significant changes in the values of CVI (0.63 ± 0.02; p=0.32), FAZ area (0.42 ± 0.13 mm^2; p=0.73) and superficial capillary plexus CDI (0.69 ± 0.08; p=0.06), and deep capillary plexus CDI (0.36 ± 0.05 p=0.72). The AVR showed a significant increase compared to baseline values (0.69 ± 0.05; p=0.03).

Multiple regression analysis was performed for the change in the choroidal thickness from baseline to 3 months based on the confounding variables of age, sex, systemic comorbidities, BMI, waist circumference, HbA1c, MAP, MOPP and the treatment received. The treatment effect was statistically significant with a p value of 0.021 after adjusting with the covariates. The mean choroidal thickness in Group A (surgery) was 61.11 µm higher than that of conservative treatment (Group B) after adjusting for the above covariates. The effect of all the covariates on the change in choroidal thickness was statistically insignificant ($p > 0.05$). A

noticeable observation was, for unit increase in HbA1c, the mean change in choroidal thickness reduced by 12.146 µm ($p=0.07$) although statistically insignificant.

Between-Group Comparison

The mean retinal and choroidal thicknesses also did not differ statistically between the two groups. There was no significant difference between the two groups in comparing the baseline and follow-up CVI, FAZ and CDI (both superficial and deep capillary plexuses), and the AVR (Table 5).

Discussion

Obesity results from morphological and functional changes in the adipose tissue, which is associated with changes in various inflammatory, hormonal, and metabolic factors [1,2,26]. Obesity can cause changes in the retinochoroidal microvasculature by a number of mechanisms, which may be the basis for ocular disease. Obesity is a multifactorial condition, and is associated with microvascular changes in several organ systems. Our study participants had a number of systemic comorbidities with obesity, demonstrating the multisystemic involvement in this condition. Increased adipose tissue in obesity has been associated with widening of venules, implicating microvascular dysfunction in the etiology of obesity [12]. Increased body weight and high BMI in obese patients can cause deranged metabolic profile due to the systemic oxidative stress secondary to hyperleptinemia [27]. Obese individuals have decreased nitric oxide (NO) levels, which results in impaired dilatation of the vessels. Also, there are increased levels of

certain vasoconstrictor molecules including endothelin-1 and angiotensin-II associated with higher BMI [3]. The choroidal blood flow is reduced through sympathetic activation and the release of noradrenalin, and increased through parasympathetic efferent nerve activation via NO signaling [28]. Lower levels of NO and increased levels of vasoconstrictor in obese patients may be responsible for decreased blood flow in the choroid. Our study supports the hypothesis that disturbance in the balance between systemic vasodilator and vasoconstrictor levels affects the choroidal blood flow.

In our study, we initially compared the retinochoroidal vascular parameters in patients with exogenous obesity with normal participants. Our study results demonstrated that patients with obesity have lower vascular retinal and choroidal vascular parameters such as CVI and CDI (Table 3). However, these participants do not have any significant difference in either the mean retinal or choroidal thickness compared to normal participants. The AVR was statistically higher in the normal subjects compared to patients with obesity (0.70 ± 0.03 versus 0.68 ± 0.05, respectively; $p<0.01$), which suggests that these subjects may have metabolic abnormalities including increased arteriolar resistance and systemic hypertension due to arteriolar narrowing. These vascular changes may affect the retinal circulation as well, demonstrated by altered AVR, resulting in reduced vascular flow of the retina and the optic nerve head. Reduced oxygenation and flow and may predispose these eyes to develop retinal vascular diseases. Thus, newer quantitative parameters on OCT and OCTA may provide important insights into the subclinical changes that occur in obese participants.

Previous studies have shown that the choroidal thickness is higher in patients with BMI between 18-24 compared to those with BMI < 18.5 (384 ± 102 µm versus 378 ± 86 µm; $p<0.01$)

[17]. Similarly, studies in obese women and children have shown that choroidal thickness is higher in patients with obesity compared to healthy control population [29,30]. In our study, we also observed higher choroidal thickness values in participants with obesity compared to healthy controls (Table 3), though it did not reach statistical significance. Indices such as CVI and CDI measured using SS-OCTA may show pathological alterations along with choroidal thickness values in obese participants. SS-OCTA has not been used previously to study these ocular parameters in obese participants. In addition, our observations on AVR are in agreement with another previous study where the values were reported to be lower than normal control participants [12]. Thus, both retinal as well as choroidal microvasculature appears to be adversely affected in obesity.

Further, we evaluated the effect of weight loss by corrective intervention (bariatric surgery or diet/exercise) on various retinochoroidal parameters. Both the groups were benefitted by intervention with a statistically significant decrease in the mean waist circumference and BMI. The change in these parameters were significantly higher after bariatric surgery compared to conservative management with diet and exercise [31]. The mean choroidal thickness increased significantly from the baseline in patients undergoing bariatric surgery. In patients receiving conservative treatment, no significant change in choroidal thickness was noted at 3 months. The increase in the choroidal thickness could be attributed to the characteristic features of choroidal flow autoregulation and the impact of systemic parameters such as MAP. In our study, we observed that MAP significantly reduced in participants of Group A following bariatric surgery (not in Group B) (Table 2; p=0.03). In the literature, studies have shown that the systemic MAP reduces both in the long-term and short-term in response to bariatric surgery [32,33]. Significant improvements occur in the hemodynamics and cardiovascular output in participants undergoing

bariatric surgery. Choroidal blood flow is significantly affected by the systemic MAP. More recent studies using animal models in the past two decades have shown that significant alterations in choroidal blood flow can occur by manipulating MAP [34–37]. Therefore, an improvement in MAP may result in higher choroidal blood flow, leading to increased choroidal thickness.

While the choroidal thickness increased significantly in participants undergoing weight reduction surgery, there was no change in the vascular parameters such as CVI, CDI and FAZ area. Both groups, however, demonstrated an improvement in the AVR but the values were statistically significant only in Group B (conservative management with diet/exercise). Improved retinal vessel AVR can result from reversal of arteriolar constriction and improved venular caliber [13].

In summary, participants with obesity have reduced retinal and choroidal vascular indices compared to normal participants. Following bariatric surgery, an increase in the choroidal thickness values and AVR may occur due to altered hemodynamics. However, parameters such as CVI and CDI may not change despite bariatric surgery. Our study has numerous limitations including a modest sample size and relatively short follow-up. Certain confounding factors could have affected the analyses in our study. Majority of the participants with obesity had co-existing systemic diseases such as diabetes and systemic hypertension. However, the multivariate regression analysis did not reveal any significant effect of the confounding factors on choroidal thickness. The Hb1Ac values did however, show an effect on choroidal thickness but it was statistically insignificant. Only 2 participants in Group A, and 3 participants in Group B did not have any comorbidities. Since this is a small number, we could not perform any meaningful

statistical subgroup analysis in participants with obesity without comorbidities. It would be ideal to include a higher sample size and participants with obesity and no vascular comorbidity. However, in the real world, since these diseases often co-exist, it is challenging to obtain such a cohort. We believe that our study also may suffer from selection bias. The prevalence of systemic comorbidities was higher in the subset of patients in Group A (those undergoing surgery) compared to Group B (managed conservatively), possibly due to higher BMI values and worse disease. However, this variability may not affect the overall comparison of patients (both groups combined) with normal participants, nor is it likely affect the before-after comparison of participants within the groups. Obesity is typically a female preponderant disease. However, in India, gender disparities and other socio-economic issues may be factors that led to a male preponderance in our cohort [38,39]. In our study, certain parameters such as AVR may not have reached statistical significance due to smaller number of participants. However, our results are in sync with previous reports and further show the utility of quantitative measurement of retinochoroidal microvasculature.

In conclusion, our study shows that SS-OCT and SS-OCTA are very useful in demonstrating subclinical changes in the retinochoroidal microvasculature in systemic diseases such as obesity, and their partial reversal following corrective surgery/therapy.

Acknowledgements

We would like to acknowledge efforts of Mr. Arun Kapil, Mr. Sushil Bhatt, and Mr. Nitin Gautam who helped in the acquisition of images in our case.

OCTA STUDY GROUP

1. Vishali Gupta (Lead of the consortium), Advanced Eye Center, Department of Ophthalmology, Post Graduate Institute of Medical Education and Research (PGIMER), Chandigarh, India. Email: vishalisara@yahoo.co.in

2. Aniruddha Agarwal, Advanced Eye Center, Department of Ophthalmology, Post Graduate Institute of Medical Education and Research (PGIMER), Chandigarh, India. Email: aniruddha9@gmail.com

3. Reema Bansal, Advanced Eye Center, Department of Ophthalmology, Post Graduate Institute of Medical Education and Research (PGIMER), Chandigarh, India. Email: drreemab@rediffmail.com

4. Rupesh Agrawal, National Healthcare Group Eye Institute, Department of Ophthalmology, Tan Tock Seng Hospital, Singapore. Email: rupeshttsh@gmail.com

5. Sarakshi Mahajan, School of Medicine, St Joseph Mercy Hospital, Oakland, Pontiac, Michigan (USA). Email: sarakshi424@gmail.com

6. Kanika Aggarwal, Advanced Eye Center, Department of Ophthalmology, Post Graduate Institute of Medical Education and Research (PGIMER), Chandigarh, India. Email: kanika2k1@yahoo.co.in

References

1. Gray DS, Fujioka K. Use of relative weight and Body Mass Index for the determination of adiposity. J Clin Epidemiol. 1991;44: 545–550. doi:10.1016/0895-4356(91)90218-x

2. Bleich S, Cutler D, Murray C, Adams A. Why is the developed world obese? Annu Rev Public Health. 2008;29: 273–295. doi:10.1146/annurev.publhealth.29.020907.090954

3. Cioffi GA, Orgül S, Onda E, Bacon DR, Van Buskirk EM. An in vivo model of chronic optic nerve ischemia: the dose-dependent effects of endothelin-1 on the optic nerve microvasculature. Curr Eye Res. 1995;14: 1147–1153. doi:10.3109/02713689508995821

4. Ferri C, Bellini C, Desideri G, Baldoncini R, Properzi G, Santucci A, et al. Circulating endothelin-1 levels in obese patients with the metabolic syndrome. Exp Clin Endocrinol Diabetes. 1997;105 Suppl 2: 38–40. doi:10.1055/s-0029-1211794

5. Cheung N, Wong TY. Obesity and eye diseases. Surv Ophthalmol. 2007;52: 180–195. doi:10.1016/j.survophthal.2006.12.003

6. Ye J, Lou L-X, He J-J, Xu Y-F. Body mass index and risk of age-related cataract: a meta-analysis of prospective cohort studies. PLoS ONE. 2014;9: e89923. doi:10.1371/journal.pone.0089923

7. Seddon JM, Cote J, Davis N, Rosner B. Progression of age-related macular degeneration: association with body mass index, waist circumference, and waist-hip ratio. Arch Ophthalmol. 2003;121: 785–792. doi:10.1001/archopht.121.6.785

8. Cheung CY, Ikram MK, Sabanayagam C, Wong TY. Retinal microvasculature as a model to study the manifestations of hypertension. Hypertension. 2012;60: 1094–1103. doi:10.1161/HYPERTENSIONAHA.111.189142

9. Rijks J, Vreugdenhil A, Dorenbos E, Karnebeek K, Joris P, Berendschot T, et al. Characteristics of the retinal microvasculature in association with cardiovascular risk markers in children with overweight, obesity and morbid obesity. Sci Rep. 2018;8: 16952. doi:10.1038/s41598-018-35279-6

10. Xiao W, Gong W, Chen Q, Ding X, Chang B, He M. Association between body composition and retinal vascular caliber in children and adolescents. Invest Ophthalmol Vis Sci. 2015;56: 705–710. doi:10.1167/iovs.14-14946

11. Siegrist M, Hanssen H, Neidig M, Fuchs M, Lechner F, Stetten M, et al. Association of leptin and insulin with childhood obesity and retinal vessel diameters. Int J Obes (Lond). 2014;38: 1241–1247. doi:10.1038/ijo.2013.226

12. Viljanen A, Soinio M, Cheung CY-L, Hannukainen JC, Karlsson HK, Wong TY, et al. Effects of bariatric surgery on retinal microvascular architecture in obese patients. Int J Obes (Lond). 2019;43: 1675–1680. doi:10.1038/s41366-018-0242-7

13. Lammert A, Hasenberg T, Kräupner C, Schnülle P, Hammes H-P. Improved arteriole-to-venule ratio of retinal vessels resulting from bariatric surgery. Obesity (Silver Spring). 2012;20: 2262–2267. doi:10.1038/oby.2012.122

14. Chhablani J, Rao PS, Venkata A, Rao HL, Rao BSK, Kumar U, et al. Choroidal thickness profi le in healthy Indian subjects. Indian J Ophthalmol. 2014;62: 1060–1063. doi:10.4103/0301-4738.146711

15. Tuncer I, Karahan E, Zengin MO, Atalay E, Polat N. Choroidal thickness in relation to sex, age, refractive error, and axial length in healthy Turkish subjects. Int Ophthalmol. 2015;35: 403–410. doi:10.1007/s10792-014-9962-4

16. Tan CS, Ouyang Y, Ruiz H, Sadda SR. Diurnal variation of choroidal thickness in normal, healthy subjects measured by spectral domain optical coherence tomography. Invest Ophthalmol Vis Sci. 2012;53: 261–266. doi:10.1167/iovs.11-8782

17. Yilmaz I, Ozkaya A, Kocamaz M, Ahmet S, Ozkaya HM, Yasa D, et al. CORRELATION OF CHOROIDAL THICKNESS AND BODY MASS INDEX. Retina (Philadelphia, Pa). 2015;35: 2085–2090. doi:10.1097/IAE.0000000000000582

18. Lavinsky F, Lavinsky D. Novel perspectives on swept-source optical coherence tomography. Int J Retina Vitreous. 2016;2: 25. doi:10.1186/s40942-016-0050-y

19. Agrawal R, Gupta P, Tan K-A, Cheung CMG, Wong T-Y, Cheng C-Y. Choroidal vascularity index as a measure of vascular status of the choroid: Measurements in healthy eyes from a population-based study. Sci Rep. 2016;6: 21090. doi:10.1038/srep21090

20. Wei X, Ting DSW, Ng WY, Khandelwal N, Agrawal R, Cheung CMG. CHOROIDAL VASCULARITY INDEX: A Novel Optical Coherence Tomography Based Parameter in Patients With Exudative Age-Related Macular Degeneration. Retina (Philadelphia, Pa). 2017;37: 1120–1125. doi:10.1097/IAE.0000000000001312

21. Agarwal A, Agrawal R, Khandelwal N, Invernizzi A, Aggarwal K, Sharma A, et al. Choroidal Structural Changes in Tubercular Multifocal Serpiginoid Choroiditis. Ocul Immunol Inflamm. 2017; 1–7. doi:10.1080/09273948.2017.1370650

22. Agrawal R, Chhablani J, Tan K-A, Shah S, Sarvaiya C, Banker A. CHOROIDAL VASCULARITY INDEX IN CENTRAL SEROUS CHORIORETINOPATHY. Retina (Philadelphia, Pa). 2016;36: 1646–1651. doi:10.1097/IAE.0000000000001040

23. Hubbard LD, Brothers RJ, King WN, Clegg LX, Klein R, Cooper LS, et al. Methods for evaluation of retinal microvascular abnormalities associated with hypertension/sclerosis in the Atherosclerosis Risk in Communities Study. Ophthalmology. 1999;106: 2269–2280. doi:10.1016/s0161-6420(99)90525-0

24. Knudtson MD, Lee KE, Hubbard LD, Wong TY, Klein R, Klein BEK. Revised formulas for summarizing retinal vessel diameters. Curr Eye Res. 2003;27: 143–149. doi:10.1076/ceyr.27.3.143.16049

25. Cheung CY-L, Hsu W, Lee ML, Wang JJ, Mitchell P, Lau QP, et al. A new method to measure peripheral retinal vascular caliber over an extended area. Microcirculation. 2010;17: 495–503. doi:10.1111/j.1549-8719.2010.00048.x

26. Dogan B, Kazim Erol M, Dogan U, Habibi M, Bulbuller N, Turgut Coban D, et al. The retinal nerve fiber layer, choroidal thickness, and central macular thickness in morbid obesity: an evaluation using spectral-domain optical coherence tomography. Eur Rev Med Pharmacol Sci. 2016;20: 886–891.

27. Manna P, Jain SK. Obesity, Oxidative Stress, Adipose Tissue Dysfunction, and the Associated Health Risks: Causes and Therapeutic Strategies. Metab Syndr Relat Disord. 2015;13: 423–444. doi:10.1089/met.2015.0095

28. Gherezghiher T, Okubo H, Koss MC. Choroidal and ciliary body blood flow analysis: application of laser Doppler flowmetry in experimental animals. Exp Eye Res. 1991;53: 151–156. doi:10.1016/0014-4835(91)90068-p

29. Bulus AD, Can ME, Baytaroglu A, Can GD, Cakmak HB, Andiran N. Choroidal Thickness in Childhood Obesity. Ophthalmic Surg Lasers Imaging Retina. 2017;48: 10–17. doi:10.3928/23258160-20161219-02

30. Yumusak E, Ornek K, Durmaz SA, Cifci A, Guler HA, Bacanli Z. Choroidal thickness in obese women. BMC Ophthalmol. 2016;16: 48. doi:10.1186/s12886-016-0227-z

31. Miras AD, le Roux CW. Mechanisms underlying weight loss after bariatric surgery. Nat Rev Gastroenterol Hepatol. 2013;10: 575–584. doi:10.1038/nrgastro.2013.119

32. Pouwels S, Lascaris B, Nienhuijs SW, Bouwman AR, Buise MP. Short-Term Changes in Cardiovascular Hemodynamics in Response to Bariatric Surgery and Weight Loss Using the Nexfin® Non-invasive Continuous Monitoring Device: a Pilot Study. Obes Surg. 2017;27: 1835–1841. doi:10.1007/s11695-017-2564-2

33. Fernstrom JD, Courcoulas AP, Houck PR, Fernstrom MH. Long-term changes in blood pressure in extremely obese patients who have undergone bariatric surgery. Arch Surg. 2006;141: 276–283. doi:10.1001/archsurg.141.3.276

34. Kur J, Newman EA, Chan-Ling T. Cellular and physiological mechanisms underlying blood flow regulation in the retina choroid in health disease. Prog Retin Eye Res. 2012;31: 377–406. doi:10.1016/j.preteyeres.2012.04.004

35. Riva CE, Titze P, Hero M, Petrig BL. Effect of acute decreases of perfusion pressure on choroidal blood flow in humans. Invest Ophthalmol Vis Sci. 1997;38: 1752–1760.

36. Polska E, Simader C, Weigert G, Doelemeyer A, Kolodjaschna J, Scharmann O, et al. Regulation of choroidal blood flow during combined changes in intraocular pressure and arterial blood pressure. Invest Ophthalmol Vis Sci. 2007;48: 3768–3774. doi:10.1167/iovs.07-0307

37. Kiel JW. Modulation of choroidal autoregulation in the rabbit. Exp Eye Res. 1999;69: 413–429. doi:10.1006/exer.1999.0717

38. Brinda EM, Attermann J, Gerdtham UG, Enemark U. Socio-economic inequalities in health and health service use among older adults in India: results from the WHO Study on Global AGEing and adult health survey. Public Health. 2016;141: 32–41. doi:10.1016/j.puhe.2016.08.005

39. Moradhvaj null, Saikia N. Gender disparities in health care expenditures and financing strategies (HCFS) for inpatient care in India. SSM Popul Health. 2019;9: 100372. doi:10.1016/j.ssmph.2019.100372

Table 1: Demographic Features of Subjects with Exogenous Obesity included in the study

Parameters	Control Subjects (n=30)	Patients		All Patients (n=60)
		Group A (n=30)	Group B (n=30)	
**Age (in years) [Mean ± SD]**	33.6 ± 8.53	42.93 ± 11.38	50.00 ± 9.26	46.47 ± 10.9
**Gender [No. (%)]**				
Male	21 (70)	24 (80)	23 (76.67)	47 (78.33)
Female	9 (30)	6 (20)	7 (23.33)	13 (21.67)
**Co-morbidities [No. (%)]**				
Diabetes	-	10 (33.33)	17 (56.67)	27 (45)
Hypertension	-	18 (60)	7 (23.33)	25 (41.67)
Osteoarthritis	-	4 (13.33)	0 (0)	4 (6.67)
Obstructive Sleep Apnea	-	8 (26.67)	1 (3.33)	9 (15)
Others	-	8 (26.67)	2 (6.67)	10 (16.67)
**Treatment of comorbidities [No. (%)]**				
Oral hypoglycemic agents	-	10 (33.33)	17 (56.67)	27 (45)
Antihypertensive agents	-	18 (60)	7 (23.33)	25 (41.67)
Lipid Lowering Agents	-	2 (6.67)	1 (3.33)	3 (5)
Others	-	6 (20)	1 (3.33)	7 (11.67)

Table 2: The baseline and follow-up systemic measurements and laboratory parameters of subjects with exogenous obesity included in the study

Parameters (Mean value) ± Standard deviation (SD)	**Group A** (n=30)			**Group B** (n=30)		
	Baseline	Follow-up	p value*	Baseline	Follow-up	p value*
Systolic blood pressure (mm Hg) ± SD	129.0 ± 7.6	127.8 ± 5.7	0.08	128.3 ± 10.1	127.6 ± 9.1	0.66
Diastolic blood pressure (mm Hg) ± SD	84.8 ± 7.2	82.6 ± 5.2	0.07	80.1 ± 7.7	80.9 ± 7.7	0.33
Mean arterial pressure ± SD	99.5 ± 6.8	97.6 ± 5.1	**0.03**	96.2 ± 7.4	96.5 ± 7.4	0.53
Weight (Kg) ± SD	119.4 ± 19.1	95.0 ± 15.2	**0.001**	86.1 ± 10.0	83.0 ± 20.7	0.46
BMI (Kg/m^2) ± SD	45.7 ± 5.3	36.5 ± 4.0	**0.001**	32.3 ± 4.3	32.1 ± 31.6	**<0.001**
Waist Circumference (cm) ± SD	105.5 ± 14.6	90.9 ± 13.3	**0.001**	92.9 ± 7.9	95.2 ± 17.7	**<0.001**
Hb1Ac (%)± SD	6.3 ± 1.4	5.5 ± 0.8	**0.001**	6.4 ± 1.4	6.1 ± 1.1	**0.02**

* Wilcoxon-Signed Rank Test

BMI: Body mass index; Hb1Ac: Glycosylated hemoglobin

Table 3: Comparison of retinochoroidal vascular parameters between normal subjects and subjects with exogenous obesity

Retinochoroidal parameters	Baseline				p value*
	Normal Subjects (n=30)		Subjects with Obesity (n=60)		
	Mean	SD	Mean	SD	
RT (µm)	279.5	28	269.23	15	0.14
CT (µm)	278.9	58	284.17	67	0.91
CVI	0.66	0.02	0.63	0.04	**<0.01**
AVR	0.7	0.04	0.68	0.05	**<0.01**
Mean FAZ area (mm^2)	0.26	0.08	0.45	0.32	**<0.01**
CDI (superficial)	0.7	0.04	0.68	0.06	**0.04**
CDI (deep)	0.38	0.02	0.35	0.06	**0.01**

* Mann-Whitney U Test

AVR: arteriovenous ratio; CDI: capillary density index; CT: choroidal thickness; CVI: choroidal vascularity index; FAZ: foveal avascular zone; RT: retinal thickness; SD: standard deviation

Table 4: Baseline and follow-up values of retinochoroidal microvasculature among subjects with exogenous obesity undergoing bariatric surgery (Group A)

Retinochoroidal parameters	**Baseline**		**3 months**		P-value*
	Mean	SD	Mean	SD	
**Group A** (n=30)					
RT (µm)	268.14	13	270.09	13	0.28
CT (µm)	272	73	329.27	79	**<0.01**
CVI	0.63	0.04	0.61	0.11	0.19
Mean FAZ area (mm^2)	0.43	0.13	0.43	0.16	0.99
AVR	0.68	0.06	0.70	0.05	0.06
CDI (superficial)	0.68	0.06	0.69	0.06	0.6
CDI (deep)	0.36	0.03	0.37	0.05	0.51

* Wilcoxon-Signed Rank Test

AVR: arteriovenous ratio; CDI: capillary density index; CT: choroidal thickness; CVI: choroidal vascularity index; FAZ: foveal avascular zone; RT: retinal thickness; SD: standard deviation

Table 5: Baseline and follow-up values of retinochoroidal microvasculature among subjects with exogenous obesity undergoing bariatric surgery (Group B)

Retinochoroidal parameters	**Baseline**		**3 months**		p value*
	Mean	SD	Mean	SD	
**Group B** (n=30)					
RT (μm)	269.99	16	271.09	16	0.32
p value**	0.74		0.56		-
CT (μm)	284.71	58	298.10	50	0.07
p value**	0.48		0.82		-
CVI	0.62	0.04	0.63	0.02	0.32
p value**	0.22		0.31		-
Mean FAZ area (mm^2)	0.48	0.43	0.42	0.13	0.73
p value**	0.88		0.78		-
AVR	0.67	0.05	0.69	0.05	**0.03**
p value**	0.82		0.56		-
CDI (superficial)	0.66	0.06	0.69	0.08	0.06
p value**	0.12		0.96		-
CDI (deep)	0.34	0.08	0.36	0.05	0.72
p value**	0.83		0.54		

* Wilcoxon-Signed Rank Test

** Mann-Whitney U Test (comparison with subjects from Group A)

AVR: arteriovenous ratio; CDI: capillary density index; CT: choroidal thickness; CVI: choroidal vascularity index; FAZ: foveal avascular zone; RT: retinal thickness; SD: standard deviation

Minimal anonymized data set

Click here to access/download
Supporting Information
Supplement data file.xls

Revised Manuscript with Track Changes

Title: Effect of w~~W~~eight l~~L~~oss on the r~~R~~etinochoroidal ~~S~~structural ~~A~~alterations ~~A~~among ~~P~~patients with ~~E~~exogenous ~~O~~obesity

Authors: Aniruddha Agarwal, MS,[1] Arshiya Saini, MBBS,[1] Sarakshi Mahajan, MD,[2] Rupesh Agrawal, FRCS,[3] Carol Y. Cheung, PhD,[4] Ashu Rastogi, MD,[5] Rajesh Gupta, MS,[6] Yu Meng Wang, PhD,[4] Michael Kwan, MD[4] Vishali Gupta, MS[1*] for the OCTA Study Group^

Affiliations:

1: Advanced Eye Center, Department of Ophthalmology, Post Graduate Institute of Medical Education and Research (PGIMER), Chandigarh, India

2: School of Medicine, St Joseph Mercy Hospital, Oakland, Pontiac, Michigan (USA)

3: National Healthcare Group Eye Institute, Department of Ophthalmology, Tan Tock Seng Hospital, Singapore

4: Department of Ophthalmology and Visual Sciences, The Chinese University of Hong Kong, Hong Kong

5: Department of Endocrinology, Post Graduate Institute of Medical Education and Research (PGIMER), Chandigarh, India

6: Department of General Surgery, Post Graduate Institute of Medical Education and Research (PGIMER), Chandigarh, India

^ Membership of the OCTA study group is provided in the acknowledgements

Short Title: Retinochoroidal vascularity in obesity

Address for CorrespondenceCorresponding Author

* Vishali Gupta, MS (

Professor of Ophthalmology

Advanced Eye Center,

Post Graduate Institute of Medical Education and Research (PGIMER)

Sector 12, Chandigarh – 160012, India

Phone: +91 172 275 6111

Fax: +91 172 274 7837

Email: vishalisara@yahoo.co.in, vishalisara@gmail.com)

SUMMARY STATEMENT

Exogenous obesity is linked to various systemic and ocular pathologies. Using swept-source optical coherence tomography and optical coherence tomography angiography, we observed altered retinochoroidal vasculature in ~~subjects~~participants with exogenous obesity compared to healthy control ~~subjects~~participants. These parameters showed improvement following interventions such as bariatric surgery.

Abstract

Purpose: To evaluate the changes in the retinochoroidal vasculature in patients with exogenous obesity using swept-source optical coherence tomography (SS-OCT) and OCT angiography (OCTA).

Methods: In this prospective study, 60 patients diagnosed with obesity (47 males) (mean age: 46.47±10.9 years) were included, of which 30 patients underwent bariatric surgery (Group A), and 30 patients underwent conservative management (exercise/diet) (Group B). Parameters including choroidal thickness (CT), choroidal vascularity index (CVI) and retinal capillary density index (CDI) and arteriovenous ratio (AVR) were measured at the baseline and three months follow up. 30 eyes (30 age and gender-matched) of normal ~~subjects~~participants were included for comparison.

Results: Baseline CT was lower in 60 ~~subjects~~participants with obesity compared to controls. Compared with normal subjects, subjects with obesity had higher mean CVI (0.66±0.02 versus 0.63±0.04; $p<0.01$), smaller FAZ area (0.26±0.07 versus 0.45±0.32; $p<0.01$), higher CDI (superficial plexus: 0.7±0.04 versus 0.68±0.06; $p=0.04$, deep plexus: 0.38±0.02 versus 0.35±0.06; $p=0.01$), and lower AVR (0.68±0.05 versus 0.70±0.03 versus; $p<0.01$). At 3-month after intervention, CT showed a significant increase in ~~subjects~~participants from Group A (329.27±79µm; $p<0.01$) but not in Group B from baseline. No significant change was noted in

CVI or CDI at 3-month in either group compared to baseline. AVR significantly increased in Group B (p=0.03).

Conclusion: Subclinical changes in retinochoroidal vasculature occurs in ~~subjects~~participants with exogenous obesity compared to healthy subjects. Surgical intervention (bariatric surgery) may have a favorable outcome on the choroidal thickness in these patients.

Keywords: Optical coherence tomography; choroidal vascularity index; obesity; bariatric surgery; choroid; perfusion

Introduction

Obesity is commonly caused by a combination of excessive food intake, lack of physical activity, and genetic susceptibility though some cases maybe be caused primarily by genetic predisposition, endocrine disorders, medications, or mental disorders, and is measured in terms of body mass index (BMI) .[1,2]. Obesity may increase levels of some vasoconstrictor molecules such as endothelin-1 and angiotensin-II [3,4] and has been linked with various ocular diseases including glaucoma, diabetic retinopathy, cataract, and age-related macular degeneration [5–7]. Therefore, obesity may be associated with alterations in the retinal microvasculature and the choroid by a number of mechanisms, which may be the basis for the various ocular diseases.

Assessment of retinal microvasculature includes measuring diameter of retinal arterioles and venules from fundus photographs .[8]. The arteriole-to-venule ratio (AVR) reflects preclinical changes in cerebral and coronary microcirculation. The AVR is calculated using the mean vessel calibre of the largest six arteries (central retinal arteriolar equivalent - CRAE) and largest six venules (central retinal venular equivalent - CRVE). In children with obesity, CRAE has been strongly linked to severe cardiovascular risk factors. [9]. Similarly, CRAE has been negatively correlated to fat mass indices in children with obesity, reflecting presence of narrow arterioles in these patients affecting the retinal microcirculation. [10]. Obesity also results in wider CRVE and lower AVR that correlate with higher leptin levels, indicating systemic metabolic abnormalities .[11]. Improved AVR has been found in patients undergone bariatric surgery after 6 months [12] and 9 months of surgery .[13].

Choroidal thickness has been found to be affected by physiological variations such as age, gender, refractive status, as well as several local and systemic diseases [14–16]. Literature shows that the choroidal thickness is lower in patients with BMI>25.0 compared to patients with BMI between 18-24 [17]. Recently, availability of swept-source optical coherence tomography (SS-OCT) and optical coherence tomography angiography (SS-OCTA) makes it possible to have precise measurements including choroidal thickness and vascular density indices [18]. The newer indicator of measuring choroidal vascularity index (CVI) indicates the ratio of choroidal vessels to stroma thus allowing the more accurate insight into the vascular alterations associated with various systemic or local diseases [19]. CVI has been reported to decrease in patients with age-related macular degeneration [20] and intraocular tuberculosis [21], while an increase is noted in central serous chorioretinopathy [22].

The alterations caused by obesity, if any, can be quantified by measuring retinal and choroidal thickness, CVI, and AVR. There is a paucity of studies in the literature that have determined the such retinochoroidal alterations in details. Moreover, the effect of correction of obesity by bariatric surgery or non-surgical measures on retinochoroidal vasculature has not been evaluated. The present study aims to describe the quantitative retinochoroidal vascular alterations associated with obesity at baseline and its correlation following an intervention (bariatric surgery or conservative management).

Materials and Methods

The index study was a prospective observational study that included 60 adult patients of either gender with exogenous obesity who presented to Department of Endocrinology, Postgraduate Institute of Medical Education and Research (PGIMER), Chandigarh. Patients who fulfilled the following inclusion criteria were enrolled in the study: Age 18-70 years, BMI≥27.5 with exogenous obesity, waist circumference of ≥80 cm, patients willing to undergo weight reduction (either medical or surgical measures). The exclusion criteria were patients with endogenous obesity (such as Cushing's syndrome), patients with retinal pathologies that can affect the retinal/choroidal thickness or vascularity such as diabetic retinopathy, macular degeneration, central serous chorioretinopathy, optic atrophy, glaucoma, and uveitis. Patients where media clarity is obscured by the presence of cataract, vitritis or any other such co-existent pathology that does not allow the acquisition of good images were also excluded. In addition, patients with refractive errors more than +3 or -3 D (high hypermetropia and myopia) were also excluded. The study was approved by the Institute Ethics Committee of PGIMER. Written informed consent was obtained from the patients enrolled in the study. The study adhered to the tenets of the Declaration of Helsinki and the provisions of the Health Insurance Portability and Accountability Act.

Baseline Evaluation

Detailed history regarding associated co-morbidities including diabetes mellitus, hypertension, obstructive sleep apnea, osteoarthritis was recorded. A detailed treatment history regarding concomitant medications being taken by the patient for several associated co-morbidities was recorded. A general physical examination was done and parameters including

height, weight, BMI, waist index, blood pressure were done. The mean arterial pressure (MAP) was also calculated. The best-corrected visual acuity (BCVA) by Snellen's chart (converted to LogMAR units for statistical analysis), intraocular pressure (IOP) measured by non-contact tonometer, slit lamp biomicroscopy for anterior segment and posterior segment examination were performed. Using the MAP and IOP values, the mean ocular perfusion pressure (MOPP) was also calculated as 2/3 of the difference between MAP and IOP.

Acquisition of Images

Thirty-degree color fundus photographs were acquired on the digital fundus camera (DRI Triton, Topcon®) with images focussed on disc and macula. SS-OCT 3D and 5-Line raster scan of the macula and optic disc were done. SS-OCTA (DRI Triton, Topcon®) using 3 × 3 scans of the macula and optic nerve head were performed. The acquisition of the scans were at least twice and better quality images were selected for further analysis. The acquisition of images was performed between 10.00 am to 12.00 noon in our study (common working hours of the Departments of Endocrinology Obesity Clinic and Ophthalmology). This also helps to minimize the effect of diurnal variations in choroidal thickness [16]. The acquired images were analyzed by three independent observers (AA, RA, and VG) and AVR, retinal and choroidal thickness, CVI, retinal thickness and retinal vascularity index were measured.

Measurement of Arteriovenous ratio

The AVR was calculated at the optic nerve head and macula using the fundus photographs. Retinal vascular caliber was measured following a standardized protocol, based on the revised Knudtson–Parr–Hubbard formula, as described in previous publications ~~.~~[23–25]. The CRAE and CRVE values were used to calculate the AVR for all the ~~subjects~~participants in the study.

Measurement of Retinal and Choroidal Thickness

Retinal thickness was measured from the inner border of the internal limiting membrane to the outer border of the retinal pigment epithelium (RPE). Choroidal thickness was measured vertically from the outer border of the RPE to the inner border of the sclera. The upper border was marked at the RPE and the lower border area was below the line of light pixels at the choroid-scleral junction. The measurements were performed at the fovea by two independent graders. The average of the two graders’ measurements was used for analysis.

Measurement of CVI

In order to measure the CVI, image binarization was performed for all the scans obtained from patients. Subfoveal scan (central B-scan) was chosen for image analyses. The image was processed on public domain software Image J (National Institutes of Health, Bethesda, USA). Polygon tool was used to select the total choroid area (TCA), which was added in the region of interest (ROI) manager. After converting the image into 8 bit, Niblack auto local thresholding

was subsequently applied which gave the mean pixel value with standard deviation for all the points. On the SS-OCT scans, the luminal Area (LA) was highlighted by applying the color threshold. In order to determine the LA within the selected polygon, both the areas in ROI manager were selected and merged by 'AND' operation of Image J. The composite third area was added to the to the ROI manager. The first area represents the total of the choroid selected, and the third composite area is the vascular or LA. Stromal area (SA), which corresponds to the interstitial or stromal component of the choroid, was obtained by calculating the differences between TCA and LA. The CVI was calculated by dividing LA by TCA.

Measurement of Retinal Capillary network

The retinal capillary network was measured in terms of foveal avascular zone (FAZ) area and capillary density index (CDI). For calculation of FAZ, manual delineation was performed on a third-party software (ImageJ). The area was calculated in square millimetres. A circle with a radius of 1.5mm is centered at the subfoveal region. Using the Niblack thresholding and ROI manager, all images were binarized and converted to 8-bits with a mean pixel value and standard deviation of all points. Subsequently, the LA was highlighted within the circle with the brightness set to 0 and 254. The LA was merged with the corresponding threshold area and measured using ROI manager. The CDI was defined as the percentage of capillary density over the stromal area within the 1.5 mm-radius circle at the macula region. CDI was obtained at both, superficial and deep retinal capillary plexus.

The patients were followed-up at 3 months following intervention (conservative management that included diet therapy and exercise, or bariatric surgery). Patients who

underwent surgery (n=30) were categorized into Group A, and patients receiving conservative therapy (n=30) were categorized to Group B. At follow-up, they underwent ocular examination, fundus photography, SS-OCT and OCTA. All the above parameters were repeated at 3 months.

We included a normative database of 30 ~~subjects~~participants from our center with no known ocular or systemic disease (mean age: 33.6 ± 8.53 years; 21 males). Similar parameters, i.e. retinal and choroidal thickness, CVI, FAZ area, CDI and AVR were calculated, and compared to patients with obesity.

Statistical Analysis

We obtained the sample size by referring to the study by Dogan et al [26] considering equal allocation ratio of 1:1, which will provide the desired effect with 95% confidence and 80% power. The statistical comparison of parameters was carried out using non-parametric tests. Multivariate regression modelling were carried out to study the effect of confounders on choroidal thickness. All the analyses were performed using SPSS version 20.0 (IBM Inc.) and the statistical significance evaluated at 5% level.

Results

Study ~~Subjects~~Participants, Demographics and Systemic Features

The study included a total of 60 patients with 30 patients in each group followed prospectively. The mean age of all patients was 46.47 ± 10.9 years (Table 1). There were a total of 47 males in the study. Both the groups matched in their demographic profile at the baseline with no statistically significant difference in any parameter. The co-morbidities of the study ~~subjects~~participants and the specific treatments are mentioned in Table 1. The mean height, weight, waist circumference, blood pressure (including MAP ~~mean arterial pressure~~), BMI, and other laboratory parameters including ~~HB1AC~~glycosylated hemoglobin (Hb1Ac) have been summarized in Table 2 (both at baseline and follow-up). At the end of 3 months, BMI was comparable in both the groups (36.50 ± 3.93 versus 39.68 ± 31.60; p=0.59). In the normal control participants, the mean height was 164.13 ± 12.3 cm, mean weight was 67.2 ± 10.64 kg (mean BMI: 24.90 ± 2.57) (p<0.001 compared to obese patients). The mean waist circumference in this group was 76.07 ± 3.76 cm (p<0.001 compared to obese patients).

In Group A, the mean BCVA was 0.007 ± 0.04 LogMAR units whereas it was 0.001 ± 0.01 LogMAR in Group B. There was no statistically significant change in the BCVA at the end of 3 months. In Group A, the MOPP was 55.78 mm Hg at baseline, and 55.01 mm Hg at 3 months (p=0.07), whereas in Group B, MOPP was 54.4 mm Hg at baseline, and 54.8 mm Hg at 3 months (p=0.47).

Comparison of Normal ~~Subjects~~Participants with Obese Patients

In comparison to the normal ~~subjects~~participants (n=30), patients with obesity (n=60) did not have statistically significant differences in either the mean retinal or choroidal thickness values at baseline. However, the mean CVI was higher among normal subjects compared to

~~subjects~~participants with obesity (0.66 ± 0.02 versus 0.63 ± 0.04, respectively; $p<0.01$). The FAZ area was significantly lower in normal ~~subjects~~participants compared to patients with obesity (0.26 ± 0.07 versus 0.45 ± 0.32, respectively; $p<0.01$). CDI was also significantly higher in normal subjects compared to obese patients (superficial plexus: 0.70 ± 0.04 versus 0.67 ± 0.06, respectively; $p=0.04$, deep plexus: 0.38 ± 0.02 versus 0.35 ± 0.06, respectively; $p=0.01$). AVR was statistically higher in the normal subjects compared to patients with obesity (0.70 ± 0.03 versus 0.68 ± 0.05, respectively; $p<0.01$).

Table 3 compares the retinochoroidal parameters between normal ~~subjects~~participants and ~~subjects~~participants with obesity.

Baseline Image Analyses among Patients with Obesity in both Groups

The baseline retinal and choroidal thicknesses in patients from Group A are listed in Table 4. The mean CVI in Group A was 0.63 ± 0.04. Analyses of SS-OCTA among ~~subjects~~participants from Group A revealed a mean FAZ area of 0.42 ± 0.13 mm^2. The mean CDI among ~~subjects~~participants in Group A was 0.68 ± 0.06 in the superficial plexus and 0.36 ± 0.03 in the deep plexus. Finally, the AVR among the patients in Group A was 0.68 ± 0.06. The baseline and follow-up values of ~~subjects~~participants in Group B are listed in Table 5. The CVI measured 0.62 ± 0.04. The mean FAZ area was 0.48 ± 0.43 mm^2. The superficial and deep capillary plexus CDI was 0.66 ± 0.06 and 0.34 ± 0.08, respectively. AVR measured 0.67 ± 0.05.

Follow-up Image Analyses among ~~Subjects~~Participants with Obesity

Follow-up values of patients of Group A and B are listed in Table 4 and 5. In Group A, the choroidal thickness showed a statistically significant increase at 3 months (329.27 ± 79; $p<0.01$). There was no significant change in the CVI among ~~subjects~~participants in Group A (0.61 ± 0.11; p=0.19). The follow-up measurements on SS-OCTA did not reveal any significant change in the FAZ area in subjects from Group A (0.43 ± 0.16 mm^2; p=0.99). Similarly, no change was noted in the CDI at 3 months in the superficial capillary plexus (0.69 ± 0.06; p=0.6) or the deep capillary plexus (0.37 ± 0.05; p=0.51). The AVR was higher at follow-up among subjects from Group A compared to baseline but not statistically significant (0.70 ± 0.05; p=0.06).

Follow-up measurements in Group B at 3 months revealed no significant change in either the retinal or choroidal thickness compared to baseline (Table 5). Similar to the observations in Group A, there were no significant changes in the values of CVI (0.63 ± 0.02; p=0.32), FAZ area (0.42 ± 0.13 mm^2; p=0.73) and superficial capillary plexus CDI (0.69 ± 0.08; p=0.06), and deep capillary plexus CDI (0.36 ± 0.05 p=0.72). The AVR showed a significant increase compared to baseline values (0.69 ± 0.05; p=0.03).

Multiple regression analysis was performed for ~~T~~the change in the choroidal thickness from baseline to 3 months ~~was regressed~~based on the confounding variables of age, sex, systemic comorbidities, BMI, waist circumference, HbA1c, MAP, MOPP~~mean arterial pressure~~ and the treatment received. The treatment effect was statistically significant with a p value of 0.021 after adjusting with the covariates. The mean choroidal thickness in Group A (surgery)

was 61.11 µm higher than that of conservative treatment (Group B) after adjusting for the above covariates. The effect of all the covariates on the change in choroidal thickness was statistically insignificant ($p > 0.05$). A noticeable observation was, for unit increase in HbA1c, the mean change in choroidal thickness reduced by 12.146 µm ($p=0.07$) although statistically insignificant.

Between-Group Comparison

-The mean retinal and choroidal thicknesses also did not differ statistically between the two groups. There was no significant difference between the two groups in comparing the baseline and follow-up CVI, FAZ and CDI (both superficial and deep capillary plexuses), and the AVR (Table 5).

Discussion

Obesity results from morphological and functional changes in the adipose tissue, which is associated with changes in various inflammatory, hormonal, and metabolic factors. [1,2,26]. Obesity can cause changes in the retinochoroidal microvasculature by a number of mechanisms, which may be the basis for ocular disease. Obesity is a multifactorial condition, and is associated with microvascular changes in several organ systems. Our study subjectsparticipants had a number of systemic comorbidities with obesity, demonstrating the multisystemic involvement in this condition. Increased adipose tissue in obesity has been associated with widening of venules, implicating microvascular dysfunction in the etiology of obesity. [12]. Increased body weight and high BMI in obese patients can cause deranged metabolic profile due to the systemic

oxidative stress secondary to hyperleptinemia. [27]. Obese individuals have decreased nitric oxide (NO) levels, which results in impaired dilatation of the vessels. Also, there are increased levels of certain vasoconstrictor molecules including endothelin-1 and angiotensin-II associated with higher BMI. [3]. The choroidal blood flow is reduced through sympathetic activation and the release of noradrenalin, and increased through parasympathetic efferent nerve activation via NO signaling. [28]. Lower levels of NO and increased levels of vasoconstrictor in obese patients may be responsible for decreased blood flow in the choroid. Our study supports the hypothesis that disturbance in the balance between systemic vasodilator and vasoconstrictor levels affects the choroidal blood flow.

In our study, we initially compared the retinochoroidal vascular parameters in patients with exogenous obesity with normal subjectsparticipants. Our study results demonstrated that patients with obesity have lower vascular retinal and choroidal vascular parameters such as CVI and CDI (Table 3). However, these subjectsparticipants do not have any significant difference in either the mean retinal or choroidal thickness compared to normal subjectsparticipants. The AVR was statistically higher in the normal subjects compared to patients with obesity (0.70 ± 0.03 versus 0.68 ± 0.05, respectively; $p<0.01$), which suggests that these subjects may have metabolic abnormalities including increased arteriolar resistance and systemic hypertension due to arteriolar narrowing. These vascular changes may affect the retinal circulation as well, demonstrated by altered AVR, resulting in reduced vascular flow of the retina and the optic nerve head. Reduced oxygenation and flow and may predispose these eyes to develop retinal vascular diseases. Thus, newer quantitative parameters on OCT and OCTA may provide important insights into the subclinical changes that occur in obese subjectsparticipants.

Previous studies have shown that the choroidal thickness is ~~lower in patients with BMI>25.0 compared to patients with~~higher in patients with BMI between 18-24 compared to those with BMI < 18.5 (384~~17~~ ± 102~~63~~ μm versus 378 ± 86 μm; p<0.01)~~.~~ [17]. Similarly, studies in obese women and children have shown that choroidal thickness is higher in patients with obesity compared to healthy control population [29,30]. In our study, we also observed higher choroidal thickness values in participants with obesity compared to healthy controls (Table 3), though it did not reach statistical significance. ~~These findings, coupled with our observations, indicate that choroidal thickness may alter only in severe cases with morbid obesity, and vascular parameters such as CVI, CDI, FAZ area, and AVR may show pathological alterations prior to any significant change in choroidal thickness compared to normal subjects.~~ Indices such as CVI and CDI measured using SS-OCTA may show pathological alterations along with choroidal thickness values in obese participants. SS-OCTA has not been used previously to study these ocular parameters in obese ~~subjects~~participants. In addition, our observations on AVR are in agreement with another previous study where the values were reported to be lower than normal control ~~subjects~~participants~~.~~ [12]. Thus, ~~B~~both retinal as well as choroidal microvasculature appears to be adversely affected in obesity.

Further, we evaluated the effect of weight loss by corrective intervention (bariatric surgery or diet/exercise) on various retinochoroidal parameters. Both the groups were benefitted by intervention with a statistically significant decrease in the mean waist circumference and BMI. The change in these parameters were significantly higher after bariatric surgery compared to conservative management with diet and exercise~~.~~ [31]. The mean choroidal thickness increased significantly from the baseline in patients undergoing bariatric surgery. ~~This was a novel finding in our study, which could indicate an improvement in the perfusion and reduction~~

~~of vasoconstriction.~~ In patients receiving conservative treatment, no significant change in choroidal thickness was noted at 3 months. The increase in the choroidal thickness could be attributed to the characteristic features of choroidal flow autoregulation and the impact of systemic parameters such as MAP. In our study, we observed that MAP significantly reduced in participants of Group A following bariatric surgery (not in Group B) (Table 2; p=0.03). In the literature, studies have shown that the systemic MAP reduces both in the long-term and short-term in response to bariatric surgery [32,33]. Significant improvements occur in the hemodynamics and cardiovascular output in participants undergoing bariatric surgery. Choroidal blood flow is significantly affected by the systemic MAP. More recent studies using animal models in the past two decades have shown that significant alterations in choroidal blood flow can occur by manipulating MAP [34–37]. Therefore, an improvement in MAP may result in higher choroidal blood flow, leading to increased choroidal thickness.

While the choroidal thickness increased significantly in ~~subjects~~participants undergoing weight reduction surgery, there was no change in the vascular parameters such as CVI, CDI and FAZ area~~, indicating that these vascular alterations may be irreversible~~. ~~This is of prognostic value, and could indicate that obesity may have long-term implications on ocular structures.~~ Both groups, however, demonstrated an improvement in the AVR but the values were statistically significant only in Group B (conservative management with diet/exercise). Improved retinal vessel AVR can result from reversal of arteriolar constriction and improved venular caliber ~~.~~[13].

In summary, ~~subjects~~participants with obesity have reduced retinal and choroidal vascular indices compared to normal ~~subjects~~participants ~~indicating suboptimal oxygenation of the tissues~~. Following bariatric surgery, ~~an improvement~~an increase in the choroidal thickness

values and AVR may ~~suggest better tissue oxygenation~~occur due to altered hemodynamics. However, ~~no change in~~parameters such as CVI and CDI may not change ~~suggest that microvascular alterations may be irreversible and may not improve~~ despite bariatric surgery~~ in patients with exogenous obesity~~. Our study has numerous limitations including a modest sample size and relatively short follow-up. Certain confounding factors could have affected the analyses in our study. Majority of the participants with obesity had co-existing systemic diseases such as diabetes and systemic hypertension. However, the multivariate regression analysis did not reveal any significant effect of the confounding factors on choroidal thickness. The Hb1Ac values did however, show an effect on choroidal thickness but it was statistically insignificant. Only 2 participants in Group A, and 3 participants in Group B did not have any comorbidities. Since this is a small number, we could not perform any meaningful statistical subgroup analysis in participants with obesity without comorbidities. It would be ideal to include a higher sample size and participants with obesity and no vascular comorbidity. However, in the real world, since these diseases often co-exist, it is challenging to obtain such a cohort. We believe that our study also may suffer from selection bias. The prevalence of systemic comorbidities was higher in the subset of patients in Group A (those undergoing surgery) compared to Group B (managed conservatively), possibly due to higher BMI values and worse disease. However, this variability may not affect the overall comparison of patients (both groups combined) with normal participants, nor is it likely affect the before-after comparison of participants within the groups. Obesity is typically a female preponderant disease. However, in India, gender disparities and other socio-economic issues may be factors that led to a male preponderance in our cohort [38,39]. In our study, ~~C~~certain parameters such as AVR may not have reached statistical significance due to smaller number of ~~subjects~~participants. However, our results are in sync with

previous reports and further show the utility of quantitative measurement of retinochoroidal microvasculature.

In conclusion, our study shows that SS-OCT and SS-OCTA are very useful in demonstrating subclinical changes in the retinochoroidal microvasculature in systemic diseases such as obesity, and their partial reversal following corrective surgery/therapy.

A. Funding/SupportAcknowledgements

None

B. Financial Disclosures

The authors have no financial disclosure/proprietary interest. The authors report no conflicts of interest. The authors alone are responsible for the content and preparation of this manuscript.

C. Other Acknowledgements

We would like to acknowledge efforts of Mr. Arun Kapil, Mr. Sushil Bhatt, and Mr. Nitin Gautam who helped in the acquisition of images in our case.

OCTA STUDY GROUP

1. Vishali Gupta (Lead of the consortium), Advanced Eye Center, Department of Ophthalmology, Post Graduate Institute of Medical Education and Research (PGIMER), Chandigarh, India. Email: vishalisara@yahoo.co.in

2. Aniruddha Agarwal, Advanced Eye Center, Department of Ophthalmology, Post Graduate Institute of Medical Education and Research (PGIMER), Chandigarh, India. Email: aniruddha9@gmail.com

3. Reema Bansal, Advanced Eye Center, Department of Ophthalmology, Post Graduate Institute of Medical Education and Research (PGIMER), Chandigarh, India. Email: drreemab@rediffmail.com

4. Rupesh Agrawal, National Healthcare Group Eye Institute, Department of Ophthalmology, Tan Tock Seng Hospital, Singapore. Email: rupeshttsh@gmail.com

5. Sarakshi Mahajan, School of Medicine, St Joseph Mercy Hospital, Oakland, Pontiac, Michigan (USA). Email: sarakshi424@gmail.com

6. Kanika Aggarwal, Advanced Eye Center, Department of Ophthalmology, Post Graduate Institute of Medical Education and Research (PGIMER), Chandigarh, India. Email: kanika2k1@yahoo.co.in

Ethics Statement

The index study was performed at the Retina and Uveitis Services of the Post Graduate Institute of Medical Education and Research, Chandigarh, India. The study was approved by the institutional review board or ethics committee. Written informed consent was obtained from the patient enrolled in the study. The study adhered to the tenets of the Declaration of Helsinki and the provisions of the Health Insurance Portability and Accountability Act.

References

1. Gray DS, Fujioka K. Use of relative weight and Body Mass Index for the determination of adiposity. J Clin Epidemiol. 1991;44: 545–550. doi:10.1016/0895-4356(91)90218-x

2. Bleich S, Cutler D, Murray C, Adams A. Why is the developed world obese? Annu Rev Public Health. 2008;29: 273–295. doi:10.1146/annurev.publhealth.29.020907.090954

3. Cioffi GA, Orgül S, Onda E, Bacon DR, Van Buskirk EM. An in vivo model of chronic optic nerve ischemia: the dose-dependent effects of endothelin-1 on the optic nerve microvasculature. Curr Eye Res. 1995;14: 1147–1153. doi:10.3109/02713689508995821

4. Ferri C, Bellini C, Desideri G, Baldoncini R, Properzi G, Santucci A, et al. Circulating endothelin-1 levels in obese patients with the metabolic syndrome. Exp Clin Endocrinol Diabetes. 1997;105 Suppl 2: 38–40. doi:10.1055/s-0029-1211794

5. Cheung N, Wong TY. Obesity and eye diseases. Surv Ophthalmol. 2007;52: 180–195. doi:10.1016/j.survophthal.2006.12.003

6. Ye J, Lou L-X, He J-J, Xu Y-F. Body mass index and risk of age-related cataract: a meta-analysis of prospective cohort studies. PLoS ONE. 2014;9: e89923. doi:10.1371/journal.pone.0089923

7. Seddon JM, Cote J, Davis N, Rosner B. Progression of age-related macular degeneration: association with body mass index, waist circumference, and waist-hip ratio. Arch Ophthalmol. 2003;121: 785–792. doi:10.1001/archopht.121.6.785

8. Cheung CY, Ikram MK, Sabanayagam C, Wong TY. Retinal microvasculature as a model to study the manifestations of hypertension. Hypertension. 2012;60: 1094–1103. doi:10.1161/HYPERTENSIONAHA.111.189142

9. Rijks J, Vreugdenhil A, Dorenbos E, Karnebeek K, Joris P, Berendschot T, et al. Characteristics of the retinal microvasculature in association with cardiovascular risk markers in children with overweight, obesity and morbid obesity. Sci Rep. 2018;8: 16952. doi:10.1038/s41598-018-35279-6

10. Xiao W, Gong W, Chen Q, Ding X, Chang B, He M. Association between body composition and retinal vascular caliber in children and adolescents. Invest Ophthalmol Vis Sci. 2015;56: 705–710. doi:10.1167/iovs.14-14946

11. Siegrist M, Hanssen H, Neidig M, Fuchs M, Lechner F, Stetten M, et al. Association of leptin and insulin with childhood obesity and retinal vessel diameters. Int J Obes (Lond). 2014;38: 1241–1247. doi:10.1038/ijo.2013.226

12. Viljanen A, Soinio M, Cheung CY-L, Hannukainen JC, Karlsson HK, Wong TY, et al. Effects of bariatric surgery on retinal microvascular architecture in obese patients. Int J Obes (Lond). 2019;43: 1675–1680. doi:10.1038/s41366-018-0242-7

13. Lammert A, Hasenberg T, Kräupner C, Schnülle P, Hammes H-P. Improved arteriole-to-venule ratio of retinal vessels resulting from bariatric surgery. Obesity (Silver Spring). 2012;20: 2262–2267. doi:10.1038/oby.2012.122

14. Chhablani J, Rao PS, Venkata A, Rao HL, Rao BSK, Kumar U, et al. Choroidal thickness profi le in healthy Indian subjects. Indian J Ophthalmol. 2014;62: 1060–1063. doi:10.4103/0301-4738.146711

15. Tuncer I, Karahan E, Zengin MO, Atalay E, Polat N. Choroidal thickness in relation to sex, age, refractive error, and axial length in healthy Turkish subjects. Int Ophthalmol. 2015;35: 403–410. doi:10.1007/s10792-014-9962-4

16. Tan CS, Ouyang Y, Ruiz H, Sadda SR. Diurnal variation of choroidal thickness in normal, healthy subjects measured by spectral domain optical coherence tomography. Invest Ophthalmol Vis Sci. 2012;53: 261–266. doi:10.1167/iovs.11-8782

17. Yilmaz I, Ozkaya A, Kocamaz M, Ahmet S, Ozkaya HM, Yasa D, et al. CORRELATION OF CHOROIDAL THICKNESS AND BODY MASS INDEX. Retina (Philadelphia, Pa). 2015;35: 2085–2090. doi:10.1097/IAE.0000000000000582

18. Lavinsky F, Lavinsky D. Novel perspectives on swept-source optical coherence tomography. Int J Retina Vitreous. 2016;2: 25. doi:10.1186/s40942-016-0050-y

19. Agrawal R, Gupta P, Tan K-A, Cheung CMG, Wong T-Y, Cheng C-Y. Choroidal vascularity index as a measure of vascular status of the choroid: Measurements in healthy eyes from a population-based study. Sci Rep. 2016;6: 21090. doi:10.1038/srep21090

20. Wei X, Ting DSW, Ng WY, Khandelwal N, Agrawal R, Cheung CMG. CHOROIDAL VASCULARITY INDEX: A Novel Optical Coherence Tomography Based Parameter in Patients With Exudative Age-Related Macular Degeneration. Retina (Philadelphia, Pa). 2017;37: 1120–1125. doi:10.1097/IAE.0000000000001312

21. Agarwal A, Agrawal R, Khandelwal N, Invernizzi A, Aggarwal K, Sharma A, et al. Choroidal Structural Changes in Tubercular Multifocal Serpiginoid Choroiditis. Ocul Immunol Inflamm. 2017; 1–7. doi:10.1080/09273948.2017.1370650

22. Agrawal R, Chhablani J, Tan K-A, Shah S, Sarvaiya C, Banker A. CHOROIDAL VASCULARITY INDEX IN CENTRAL SEROUS CHORIORETINOPATHY. Retina (Philadelphia, Pa). 2016;36: 1646–1651. doi:10.1097/IAE.0000000000001040

23. Hubbard LD, Brothers RJ, King WN, Clegg LX, Klein R, Cooper LS, et al. Methods for evaluation of retinal microvascular abnormalities associated with hypertension/sclerosis in the Atherosclerosis Risk in Communities Study. Ophthalmology. 1999;106: 2269–2280. doi:10.1016/s0161-6420(99)90525-0

24. Knudtson MD, Lee KE, Hubbard LD, Wong TY, Klein R, Klein BEK. Revised formulas for summarizing retinal vessel diameters. Curr Eye Res. 2003;27: 143–149. doi:10.1076/ceyr.27.3.143.16049

25. Cheung CY-L, Hsu W, Lee ML, Wang JJ, Mitchell P, Lau QP, et al. A new method to measure peripheral retinal vascular caliber over an extended area. Microcirculation. 2010;17: 495–503. doi:10.1111/j.1549-8719.2010.00048.x

26. Dogan B, Kazim Erol M, Dogan U, Habibi M, Bulbuller N, Turgut Coban D, et al. The retinal nerve fiber layer, choroidal thickness, and central macular thickness in morbid obesity: an evaluation using spectral-domain optical coherence tomography. Eur Rev Med Pharmacol Sci. 2016;20: 886–891.

27. Manna P, Jain SK. Obesity, Oxidative Stress, Adipose Tissue Dysfunction, and the Associated Health Risks: Causes and Therapeutic Strategies. Metab Syndr Relat Disord. 2015;13: 423–444. doi:10.1089/met.2015.0095

28. Gherezghiher T, Okubo H, Koss MC. Choroidal and ciliary body blood flow analysis: application of laser Doppler flowmetry in experimental animals. Exp Eye Res. 1991;53: 151–156. doi:10.1016/0014-4835(91)90068-p

29. Bulus AD, Can ME, Baytaroglu A, Can GD, Cakmak HB, Andiran N. Choroidal Thickness in Childhood Obesity. Ophthalmic Surg Lasers Imaging Retina. 2017;48: 10–17. doi:10.3928/23258160-20161219-02

30. Yumusak E, Ornek K, Durmaz SA, Cifci A, Guler HA, Bacanli Z. Choroidal thickness in obese women. BMC Ophthalmol. 2016;16: 48. doi:10.1186/s12886-016-0227-z

31. Miras AD, le Roux CW. Mechanisms underlying weight loss after bariatric surgery. Nat Rev Gastroenterol Hepatol. 2013;10: 575–584. doi:10.1038/nrgastro.2013.119

32. Pouwels S, Lascaris B, Nienhuijs SW, Bouwman AR, Buise MP. Short-Term Changes in Cardiovascular Hemodynamics in Response to Bariatric Surgery and Weight Loss Using the Nexfin® Non-invasive Continuous Monitoring Device: a Pilot Study. Obes Surg. 2017;27: 1835–1841. doi:10.1007/s11695-017-2564-2

33. Fernstrom JD, Courcoulas AP, Houck PR, Fernstrom MH. Long-term changes in blood pressure in extremely obese patients who have undergone bariatric surgery. Arch Surg. 2006;141: 276–283. doi:10.1001/archsurg.141.3.276

34. Kur J, Newman EA, Chan-Ling T. Cellular and physiological mechanisms underlying blood flow regulation in the retina choroid in health disease. Prog Retin Eye Res. 2012;31: 377–406. doi:10.1016/j.preteyeres.2012.04.004

35. Riva CE, Titze P, Hero M, Petrig BL. Effect of acute decreases of perfusion pressure on choroidal blood flow in humans. Invest Ophthalmol Vis Sci. 1997;38: 1752–1760.

36. Polska E, Simader C, Weigert G, Doelemeyer A, Kolodjaschna J, Scharmann O, et al. Regulation of choroidal blood flow during combined changes in intraocular pressure and arterial blood pressure. Invest Ophthalmol Vis Sci. 2007;48: 3768–3774. doi:10.1167/iovs.07-0307

37. Kiel JW. Modulation of choroidal autoregulation in the rabbit. Exp Eye Res. 1999;69: 413–429. doi:10.1006/exer.1999.0717

38. Brinda EM, Attermann J, Gerdtham UG, Enemark U. Socio-economic inequalities in health and health service use among older adults in India: results from the WHO Study on Global AGEing and adult health survey. Public Health. 2016;141: 32–41. doi:10.1016/j.puhe.2016.08.005

39. Moradhvaj null, Saikia N. Gender disparities in health care expenditures and financing strategies (HCFS) for inpatient care in India. SSM Popul Health. 2019;9: 100372. doi:10.1016/j.ssmph.2019.100372

Table 1: Demographic Features of Subjects with Exogenous Obesity included in the study

Parameters	Control Subjects (n=30)	Patients Group A (n=30)	Patients Group B (n=30)	All Patients (n=60)
**Age (in years) [Mean ± SD]**	33.6 ± 8.53	42.93 ± 11.38	50.00 ± 9.26	46.47 ± 10.9
**Gender [No. (%)]**				
Male	21 (70)	24 (80)	23 (76.67)	47 (78.33)
Female	9 (30)	6 (20)	7 (23.33)	13 (21.67)
**Co-morbidities [No. (%)]**				
Diabetes	-	10 (33.33)	17 (56.67)	27 (45)
Hypertension	-	18 (60)	7 (23.33)	25 (41.67)
Osteoarthritis	-	4 (13.33)	0 (0)	4 (6.67)
Obstructive Sleep Apnea	-	8 (26.67)	1 (3.33)	9 (15)
Others	-	8 (26.67)	2 (6.67)	10 (16.67)
**Treatment of comorbidities [No. (%)]**				
Oral hypoglycemic agents	-	10 (33.33)	17 (56.67)	27 (45)
Antihypertensive agents	-	18 (60)	7 (23.33)	25 (41.67)
Lipid Lowering Agents	-	2 (6.67)	1 (3.33)	3 (5)
Others	-	6 (20)	1 (3.33)	7 (11.67)

Table 2: The baseline and follow-up systemic measurements and laboratory parameters of subjects with exogenous obesity included in the study

Parameters (Mean value) ± Standard deviation (SD)	**Group A** (n=30)			**Group B** (n=30)		
	Baseline	Follow-up	p value*	Baseline	Follow-up	p value*
Systolic blood pressure (mm Hg) ± SD	129.0 ± 7.6	127.8 ± 5.7	0.08	128.3 ± 10.1	127.6 ± 9.1	0.66
Diastolic blood pressure (mm Hg) ± SD	84.8 ± 7.2	82.6 ± 5.2	0.07	80.1 ± 7.7	80.9 ± 7.7	0.33
Mean arterial pressure ± SD	99.5 ± 6.8	97.6 ± 5.1	**0.03**	96.2 ± 7.4	96.5 ± 7.4	0.53
Weight (Kg) ± SD	119.4~~0~~ ± 19.1	95.0~~0~~ ± 15.2	**0.001**	86.1~~3~~ ± 10.0	83.0~~1~~ ± 20.7	0.46
BMI (Kg/m^2) ± SD	45.7~~69~~ ± 5.3	36.5~~0~~ ± 4.0	**0.001**	32.3~~0~~ ± 4.3	32.1~~1~~ ± 31.6	**<0.001**
Waist Circumference (cm) ± SD	105.5~~47~~ ± 14.6	90.9~~1~~ ± 13.3	**0.001**	92.9~~86~~ ± 7.9	95.2~~2~~ ± 17.7	**<0.001**
~~HB1AC~~Hb1Ac (%)± SD	6.3~~25~~ ± 1.4	5.5~~48~~ ± 0.8	**0.001**	6.4~~39~~ ± 1.4	6.1~~09~~ ± 1.1	**0.02**

* Wilcoxon-Signed Rank Test

BMI: Body mass index; ~~HB1AC~~Hb1Ac: ~~Hemoglobin A1C~~Glycosylated hemoglobin

Table 3: Comparison of retinochoroidal vascular parameters between normal subjects and subjects with exogenous obesity

Retinochoroidal parameters	Baseline				p value*
	Normal Subjects (n=30)		Subjects with Obesity (n=60)		
	Mean	SD	Mean	SD	
RT (µm)	279.5	28	269.23	15	0.14
CT (µm)	278.9	58	284.17	67	0.91
CVI	0.66	0.02	0.63	0.04	**<0.01**
AVR	0.7	0.04	0.68	0.05	**<0.01**
Mean FAZ area (mm^2)	0.26	0.08	0.45	0.32	**<0.01**
CDI (superficial)	0.7	0.04	0.68	0.06	**0.04**
CDI (deep)	0.38	0.02	0.35	0.06	**0.01**

* Mann-Whitney U Test

AVR: arteriovenous ratio; CDI: capillary density index; CT: choroidal thickness; CVI: choroidal vascularity index; FAZ: foveal avascular zone; RT: retinal thickness; SD: standard deviation

Table 4: Baseline and follow-up values of retinochoroidal microvasculature among subjects with exogenous obesity undergoing bariatric surgery (Group A)

Retinochoroidal parameters	**Baseline**		**3 months**		P-value*
	Mean	SD	Mean	SD	
**Group A** (n=30)					
RT (μm)	268.14	13	270.09	13	0.28
CT (μm)	272	73	329.27	79	**<0.01**
CVI	0.63	0.04	0.61	0.11	0.19
Mean FAZ area (mm^2)	0.43	0.13	0.43	0.16	0.99
AVR	0.68	0.06	0.70	0.05	0.06
CDI (superficial)	0.68	0.06	0.69	0.06	0.6
CDI (deep)	0.36	0.03	0.37	0.05	0.51

* Wilcoxon-Signed Rank Test

AVR: arteriovenous ratio; CDI: capillary density index; CT: choroidal thickness; CVI: choroidal vascularity index; FAZ: foveal avascular zone; RT: retinal thickness; SD: standard deviation

Table 5: Baseline and follow-up values of retinochoroidal microvasculature among subjects with exogenous obesity undergoing bariatric surgery (Group B~~A~~)

Retinochoroidal parameters	**Baseline**		**3 months**		p value*
	Mean	SD	Mean	SD	
**Group B** (n=30)					
RT (µm)	269.99	16	271.09	16	0.32
p value**	0.74		0.56		-
CT (µm)	284.71	58	298.10	50	0.07
p value**	0.48		0.82		-
CVI	0.62	0.04	0.63	0.02	0.32
p value**	0.22		0.31		-
Mean FAZ area (mm^2)	0.48	0.43	0.42	0.13	0.73
p value**	0.88		0.78		-
AVR	0.67	0.05	0.69	0.05	**0.03**
p value**	0.82		0.56		-
CDI (superficial)	0.66	0.06	0.69	0.08	0.06
p value**	0.12		0.96		-
CDI (deep)	0.34	0.08	0.36	0.05	0.72
p value**	0.83		0.54		

* Wilcoxon-Signed Rank Test

** Mann-Whitney U Test (comparison with subjects from Group A)

AVR: arteriovenous ratio; CDI: capillary density index; CT: choroidal thickness; CVI: choroidal vascularity index; FAZ: foveal avascular zone; RT: retinal thickness; SD: standard deviation

May 11, 2020

Pukhraj Rishi, MD
Academic Editor
PLOS ONE

RE: PONE-D-20-08014: Effect of Weight Loss on the Retinochoroidal Structural Alterations Among Patients with Exogenous Obesity

Dear Dr. Rishi:

On behalf of all the authors, I wish to thank you and the reviewers helping us improve our manuscript. We appreciate the comments and have addressed them as indicated below.

Journal Requirements:
1. Please ensure that your manuscript meets PLOS ONE's style requirements, including those for file naming. The PLOS ONE style templates can be found at https://journals.plos.org/plosone/s/file?id=wjVg/PLOSOne_formatting_sample_main_body.pdf and https://journals.plos.org/plosone/s/file?id=ba62/PLOSOne_formatting_sample_title_authors_affiliations.pdf
Response 1: Thank you very much for providing the links to the PLOS ONE style templates. We have incorporated these in the revised manuscript.

2. Thank you for stating the following financial disclosure:
"The funders had no role in study design, data collection and analysis, decision to publish, or preparation of the manuscript"
At this time, please address the following queries:
a) Please clarify the sources of funding (financial or material support) for your study. List the grants or organizations that supported your study, including funding received from your institution.
b) State what role the funders took in the study. If the funders had no role in your study, please state: “The funders had no role in study design, data collection and analysis, decision to publish, or preparation of the manuscript.”
c) If any authors received a salary from any of your funders, please state which authors and which funders.
d) If you did not receive any funding for this study, please state: “The authors received no specific funding for this work.”
Please include your amended statements within your cover letter; we will change the online submission form on your behalf.
Response 2: We have noted the above instructions. We have not received any funding for this work. The statement, “The authors received no specific funding for this work” has been added to the Cover Letter. Thank you.

3. One of the noted authors is a group or consortium 'OCTA Study Group'. In addition to

naming the author group, please list the individual authors and affiliations within this group in the acknowledgments section of your manuscript. Please also indicate clearly a lead author for this group along with a contact email address.

Response 3: Thank you very much for the comment. As per the journal requirements, we have stated the details of the OCTA study group in the acknowledgements section of the manuscript. Individual email addresses are also listed.

4. Your ethics statement must appear in the Methods section of your manuscript. If your ethics statement is written in any section besides the Methods, please move it to the Methods section and delete it from any other section. Please also ensure that your ethics statement is included in your manuscript, as the ethics section of your online submission will not be published alongside your manuscript.

Response 4: Thank you very much for the comment. We have added the following statement in the methods section, "The study was approved by the Institute Ethics Committee of PGIMER and adhered to the tenets of Declaration of Helsinki."

Page 8, First Paragraph

Additional Editor Comments:

The reviewers have raised several pertinent concerns including those related to the methodology of the study. However, in view of the interesting research question, it is worthwhile to have the authors an opportunity to revise the manuscript.

Response: We would like to thank the Additional Editor for thoroughly reviewing our manuscript, and giving us the chance to respond to the reviewer's comments. We have significantly modified the manuscript based on the comments, clarified several aspects of the methodology, and improved reporting of the results. We hope that the revisions are appropriate, and the manuscript can be considered further.

Reviewer #1:

1. Table 1 details baseline characteristics of Groups A and B but not the normal subjects.

Response 1: Thank you very much for the comment. We have included baseline characters of normal control population in Table 1.

2. Table 2 does not give the standard deviations or confidence intervals.

Response 2: Thank you very much for the suggestions. We have included the standard deviation values in the Table 2.

3. The authors defined the obesity as BMI>27.5 and waist circumference of more than 80 cm. However, the authors do not give the actual distribution and comparison of the BMI and waist circumference between normal and obese subjects in this study. This may be important to make sure that the normal subjects are really normal.

Response 3: Thank you very much for highlighting this important aspect. We have mentioned the height, weight, BMI and waist circumference of the normal

control subjects included in this study in the results section. The normal subjects had a mean BMI of 24.90 and mean waist circumference of 76.07 cm.

Results section; Page 13, 1st paragraph

4. The methodology does not specify where the choroidal thickness and retinal thickness were measured - in relation to fovea and whether the measurement taken for analysis is an average of several measurements?
Response 4: Thank you once again for highlighting this pertinent aspect. We have added the missing information in the methods. The measurements were performed at the fovea. The following statement has been added, "The measurements were performed at the fovea by two independent graders. The average of the two graders' measurements was used for analysis."

Methods section; Page 10; 2nd paragraph

5. One is not clear also whether the measurements within one subject were taken at roughly the same time of the day? There are studies to show significant diurnal variation in choroidal thickness of up to 33.7 micrometers (Tan CS, Ouyang Y, Ruiz H, Sadda SR. Diurnal variation of choroidal thickness in normal, healthy subjects measured by Spectral domain optical coherence tomography. IOVS 2012;53:261-266)
Response 5: As the reviewer suggested, it is important to keep in mind the diurnal variation of the choroidal thickness and therefore, all measurements need to be performed at a particular time interval in the day. In our study, all the measurements were performed between 10.00 am to 12.00 noon. This is also the common working hours of the Obesity Clinic and Ophthalmology Clinic in our institute. We have mentioned this information in the revised manuscript.

Page 9; Section on "Acquisition of Images"

6. Are there any obese subjects without comorbidities? They would form a good subgroup for analysis of effect of obesity itself (if any) on the parameters studied.
Response 6: In our cohort, among the patients in Group A (those undergoing surgery), there were only 2 patients who did not have additional co-morbidities. Likewise, in Group B (those receiving conservative therapy), 3 patients did not have any additional co-morbidities. Therefore, we believe that this is a small number that may not allow meaningful statistical analysis. Unfortunately, subjects with obesity tend to have various comorbidities, and this has been mentioned in the limitations of the manuscript.

Page 19, Last line; Page 20, first 3 lines

7. To evaluate the role of coexisting vascular disorder versus only obesity on the vascular parameters that have been studied, one would ideally need two controls- a) non-obese subjects with systemic vascular disorder (roughly of the same duration and severity as seen in the obese subjects. And b) as mentioned above- any subgroup of

obese subjects without vascular comorbidity (perhaps such a group may be difficult to get).
Response 7: Thank you very much for the insightful comment. We agree with the reviewer that it would be ideal to have two such groups – one of non-obese subjects with systemic vascular diseases of same duration and severity, and obese subjects without vascular comorbidity. But as the reviewer suggests, in the real world, it is very difficult to obtain such a cohort especially because some of these diseases co-exist. We have added this factor in the discussion, highlighting the limitations of our selection.

Page 20; First paragraph

8. The authors conclude – 'While the choroidal thickness increased significantly in subjects undergoing weight reduction surgery, there was no change in the vascular parameters such as CVI, CDI and FAZ area, indicating that these vascular alterations may be irreversible.' There are several issues to this sweeping conclusion.
a) At baseline the authors found choroidal thickness and retinal thickness to be similar between normal subjects and obese subjects. But the obese subjects had increased choroidal thickness after bariatric surgery - does this mean their choroidal thickness is now supra normal?
b) The other vascular parameters - CVI, AVR, FAZ, CDI have not altered before and after. To conclude this- as indicative of permanent damage to vessels would also force one to admit that the choroidal thickness becomes supra normal after bariatric surgery!
c) The authors excluded all patients with obvious retinal vascular disorder among the subjects with obesity. Hence it is understandable that the systemic vascular disease is also likely to be of short duration and perhaps under adequate control.
Under such circumstances it would be difficult to accept a conclusion that attributes the lack of difference in the parameters before and after weight reduction to irreversible vascular changes.
Response 8: Thank you very much for the comments. We agree with your observations and accordingly, have performed additional literature search to understand the choroidal flow and autoregulation in systemic diseases. In response to point b, we also calculated Mean ocular perfusion pressure (MOPP) as one of the vascular flow regulatory factor in the ocular circulation and possibly affecting CVI, AVR and CDI. The revised manuscript now provides an explanation as to why the choroidal thickness could have increased following surgery.

We observed an interesting finding in our study. The mean arterial pressure (MAP) significantly reduced in Group A (those subjects undergoing surgery) and not in Group B (patients managed conservatively). However, there was no statistically significant difference in MOPP in both the groups. This has been added to the revised Table 2. Studies have shown that the systemic MAP reduces both in the long-term and short-term in response to bariatric surgery. Choroidal blood flow is significantly affected by MAP, and therefore, it may result in increased choroidal thickness postoperatively in patients of Group A. Hence, the choroidal thickness should not be labeled as 'supra normal'.

Among the other vascular parameters, CVI and CDI were lower in obese subjects compared to normal healthy controls. However, we have removed the argument that change in CVI and CDI indicates permanent damage to these structures.

Page 19, multiple places

Reviewer #2:
1. The comparison of baseline features in subjects versus controls is skewed by significant prevalence of diabetes and hypertension, etc. which are known to affect choroidal vasculature and FAZ. The groups A, B are different systemically - when systemic disease and treatment is being studied for its effect on an ocular finding, we expect the groups to be better matched.
Response 1: We would like to thank the reviewer for the insightful comments. The baseline comorbidities are different among subjects in Groups A and B. This is likely due to a selection bias, i.e. subjects who are in Group A and undergoing bariatric surgery may be the ones with higher BMI (as reflected in Table 2), and with higher BMI, complications and comorbidities such as hypertension and sleep apnea can be higher than those in Group B (patients not undergoing surgery). Therefore, this has been mentioned in the revised manuscript in the limitations.
However, the variability of the comorbidities and systemic disease between Group A and Group B will not affect the overall comparison of patients (both groups combined) with normal subjects, nor will it affect the before-after comparison of subjects within the groups. Therefore, we believe that our data set is still pertinent and with the inherent limitations, comparison between the groups is still possible. Thank you very much.

Page 20; Discussion

2. Also, we do not know what happened to the systemic disease after bariatric surgery or weight reduction - did it affect blood pressures, DM control, etc?
Response 2: Thank you very much for the comment. We have already reported the follow-up changes in the weight, BMI, waist circumference, and HB1AC in patients from Group A and B in Table 2. Additionally, we have added the mean values of systolic and diastolic blood pressures in the two groups at baseline and follow-up in Table 2. We also calculated and added the systemic mean arterial pressure (MAP) in Table 2, and realized that subjects undergoing bariatric surgery (Group A) had a significantly reduced MAP in the follow-up period.

In the follow-up, the subjects also experienced a statistically significant improvement in parameters such as weight (in Group A), BMI, waist circumference and HB1AC. However, there was no statistically significant difference in the mean systolic and diastolic blood pressure. This information is provided in Table 2.

3. We know that HTN/ DM get better with weight reduction and more so with bariatric surgery... so the systemic status of the groups and changes in medication after the intervention will be useful to know, and any meaningful conclusion can only be derived from that.
Response 3: Thank you very much for the comment. In Group A (the subjects who underwent bariatric surgery), the systolic and diastolic blood pressure improved at follow-up, but did not reach statistical significance. However, there was improvement in diabetes (reflected by HB1AC values). Thank you very much for the suggestion.

Results; Table 2

4. Where there are so many systemic variables confounding the basic disease (Obesity), the study would need a much larger sample size to have relevant statistics.
Response 4: The sample size has been calculated for the study based on the study by Dogan et al [26] considering equal allocation ratio of 1:1, which will provide the desired effect with 95% confidence and 80% power. As per Dogan et al, the choroidal thickness for obese group was 300.63 ± 65.55 µm, while that of normal group was 338.79 ± 64.41 µm. Accordingly, the effect size obtained was 0.59 (~0.60). In the current study, a sample size of in each group was obtained as 30. However, we do agree that a larger data set would help us understand the changes in the parameters better. Large dataset is challenging to obtain, since the number of patients undergoing bariatric surgery may be less. Hence, in the discussion, we have stated that one of the limitations of our study is the modest sample size.

Methods Section; Page 12; 2nd paragraph

Reviewer #3:
1. Obesity is a female preponderant disease. How do you explain male preponderance in your cases? Was diurnal variation accounted for while measuring choroidal thickness? Co-morbidities are mentioned at baseline but not at final follow up. What improvements were noted in co-morbid factors like BP, Hb AIC at final follow up and their effect on final CT and other variables?
Response 1: Thank you very much for highlighting these pertinent aspects. We agree that obesity is a female preponderant disease. However, in India, gender disparities in health care expenditures are well known due to various socioeconomic factors. Studies have shown that the health care expenditures on adult Indian females is systemically lower than that of adult males. Such socioeconomic disparities in health and health care are major concerns in India. We have added these references and the possible selection bias in the limitations.
We performed the OCT scans between 10.00 am to 12.00 noon in our study to minimize the effect of diurnal variations (explained in the response to reviewer #1, comment 5).

We have now mentioned the comorbidities at follow-up as well. The improvements in HB1AC, BP and mean arterial pressure has been added to the revised manuscript.

Page 20; Discussion; Table 2; multiple places

2. CT depends on multitude of factors. Was multivariate analysis performed to look for the effect of other variables? Kindly provide the data

Response 2: Thank you very much for raising this very valid point. We have also added Mean ocular perfusion pressure (MOPP) in addition to all the possible factors which can affect CT and other vascular parameters in the eye. We have included the multiple regression analysis in the revised results. The multiple regression reveals that none of the confounders had a significant impact on the change in choroidal thickness from baseline to 3 months. It is noteworthy that with every unit increase in HB1AC, there was a change in the choroidal thickness of approximately 12 microns, but this was not statistically significant. A limitation, therefore, has been added to the discussion that a higher sample would be better for a detailed analysis.
An interesting aspect was that the only variable associated with higher choroidal thickness on multivariate analysis was the treatment strategy (significantly higher in those undergoing bariatric surgery).

Page 15; paragraph 3

3. Review of literature reveals that CT is higher in patients with obesity. However the present study shows decreased CT in patients with obesity. This appears to be major conflicting result vis a vis published literature. Authors need to clarify this point in a more robust way in review of literature

1. Yilmaz I, Ozkaya A, Kocamaz M, et al. CORRELATION OF CHOROIDAL THICKNESS AND BODY MASS INDEX. Retina (Philadelphia, Pa). 2015;35(10):2085-2090. doi:10.1097/IAE.0000000000000582

2. Ophthalmic Surg Lasers Imaging Retina. 2017 Jan 1;48(1):10-17. doi: 10.3928/23258160-20161219-02. Choroidal Thickness in Childhood Obesity. Bulus AD, Can ME, Baytaroglu A, Can GD, Cakmak HB, Andiran N.

3. BMC Ophthalmol. 2016 May 4;16(1):48. doi: 10.1186/s12886-016-0227-z. Choroidal thickness in obese women. Yumusak E1, Ornek K2, Durmaz SA3, Cifci A4, Guler HA2, Bacanli Z4.

Response 3: Thank you very much for the comment. We agree that the previous studies listed by the reviewer have reported a higher choroidal thickness in subjects with obesity compared to healthy controls. In our study, the choroidal thickness in normal subjects was 278.9 microns, and 284.2 microns in subjects with obesity. Thus, we also found higher choroidal thickness values compared to normal subjects, but this did not reach statistical significance.

The manuscript by Yilmaz et al showed that subjects with BMI between 18.5-24.9 have higher choroidal thickness than those with BMI > 25. In the study by Bulus et al, the choroidal thickness was higher in obese children. Yumusak et al have also shown higher choroidal thickness values in obese women compared to control population. The revised statements and newer references have been added in the revised manuscript.

Page 17; Paragraph 2

Author Changes
We have added the values for mean ocular perfusion pressure (MOPP) calculated from mean arterial pressure and IOP. This has been mentioned in the methods (Page 9; first paragraph) and results (Page 13; 2nd paragraph).

We hope our responses will satisfy the reviewers. We thank the Editor and the reviewers for dedicating their time and effort to review our manuscript. We hope that the manuscript is now appropriate for publication in *Retina*.

Sincerely,

Vishali Gupta, MS for the authors
