# Supplementary material for: Effect of weight loss on the retinochoroidal structural alterations among patients with exogenous obesity
Source: PLoS One. 2020 Jul 9;15(7):e0235926. doi: 10.1371/journal.pone.0235926 (PMC7347179; doi:10.1371/journal.pone.0235926)
Supplement: Attachment — Submitted filename: Comments from Reviewers_AA_06192020.docx [file pone.0235926.s004.docx]

**June 20, 2020**

**Pukhraj Rishi, MD**

**Academic Editor**

***PLOS ONE***

**RE: PONE-D-20-08014R1: Effect of Weight Loss on the Retinochoroidal Structural Alterations Among Patients with Exogenous Obesity**

**Dear Dr. Rishi:**

**On behalf of all the authors, I wish to thank you and the reviewers helping us improve our manuscript. We appreciate the comments and have addressed them as indicated below.**

**Additional Editor Comments:**
1. Authors have quoted a study of normative data based on imaging of 81 eyes with SDOCT, while their own study was performed with SSOCT. Suggest reference to another study on normative data based on imaging of 230 eyes with SSOCT may be more appropriate. Akhtar Z, Rishi P, Srikanth R, Rishi E, Bhende M, Raman R. Choroidal thickness in normal Indian subjects using Swept source optical coherence tomography. PLoS One. 2018;13(5):e0197457. Published 2018 May 16. doi:10.1371/journal.pone.0197457

**Response: Thank you very much for the suggestion. We have added the suggested reference to the manuscript.**

**New reference 17**

2. If all the patients were recruited from PGI Chandigarh then how is HIPAA relevant in India?

**Response: This statement has been removed from the manuscript.**

**Page 8, 1^st^ Paragraph, Last line**

3. Rephrase this sentence (The composite third area was added to the to the ROI manager.

**Response: The statement has been corrected. Thank you.**

**Page 11, 1^st^ Paragraph, 3^rd^ Line**

4. The title of the Table represents data from group B but seems to indicate the cohort underwent bariatric surgery!

**Response: We apologize for the error. The table 5 represents data from Group B, i.e. patients receiving conservative management.**

**Table 5**

5. “Our study supports the hypothesis that disturbance in the balance between systemic vasodilator and vasoconstrictor levels affects the choroidal blood flow.”

How? This is a broad statement and the study is not designed to answer this question. You might be asked to substantiate with biochemical parameters.

**Response: We agree with the reviewer, and this statement has been removed from the manuscript.**

**Page 17, 1^st^ Paragraph, Last 3 Lines**

6. “Both the groups were benefitted by intervention with a statistically significant decrease in the mean waist circumference and BMI.”

On the contrary the waist circumference increased in group B (Table 2). Please revise the statement, and how do you explain the difference in the two groups.

**Response: Thank you very much for the comment. We have revised the statement as “Both the groups were benefitted by intervention with a statistically significant decrease in the mean waist circumference (in Group A) and BMI (in both the groups) (Table 2).” One explanation of this phenomenon could be an error in the measurements of the waist.**

**Page 18, 2^nd^ Paragraph, Lines 4-5**

7. It was interesting to note that in group A, post-surgery, CVI decreased although not in a statistically significant way. So, we need to continue to follow-up these patients for a better understanding.

**Response: We agree with the reviewer. We shall continue long-term follow-up of these patients. Thank you very much.**

8. Mean age was much younger than Obesity groups. Any comments?

**Response: Thank you very much for the comment. The mean age difference between controls and subjects in our study was approximately 12.8 years. Based on the previous publications, the choroidal thickness is reduced by approximately 17 µm per decade after 30 years of age (Akhtar et al). This limitation has been provided in the manuscript. However, Agrawal et al have shown that on multiple regression model, CVI is not statistically associated with age. This has been added to the revised manuscript.**

**Page 20, 1^st^ Paragraph.**

**We hope our responses will satisfy the reviewers. We thank the Editor and the reviewers for dedicating their time and effort to review our manuscript. We hope that the manuscript is now appropriate for publication in *Retina*.**

**Sincerely,**

**Vishali Gupta, MS for the authors**
